# Explainable machine learning for breast cancer prediction in resource-constrained settings: A multi-algorithmic framework integrating shap-based transparency with clinical decision support

**DOI:** 10.1371/journal.pdig.0001706

**Published:** 2026-09-11

**Authors:** Oluwaseun Adebayo Bamodu, Sumaiya Nezam, Chen-Chih Chung

**Affiliations:** 1 Building Research and Implementation to Drive Growth and Equity in Africa (BRIDGEAfrica), Dar Es Salaam, Tanzania; 2 Bethesda Achievers Consultancy, Frederick, Maryland, United States of America; 3 Department of Prevention and Community Health, Milken Institute School of Public Health, The George Washington University, Washington, District of Columbia, United States of America; 4 Department of Neurology, Taipei Medical University-Shuang Ho Hospital, New Taipei City, Taiwan; 5 Department of Neurology, School of Medicine, College of Medicine, Taipei Medical University, Taipei City, Taiwan; 6 Taipei Neuroscience Institute, Taipei Medical University-Shuang Ho Hospital, New Taipei City, Taiwan; National Taichung University of Science and Technology, TAIWAN

## Abstract

Breast cancer remains the most commonly diagnosed malignancy among women globally, with disproportionately higher mortality rates in low- and middle-income countries (LMICs) where diagnostic delays and limited specialist pathology capacity are widespread. While machine learning (ML) approaches achieve strong predictive performance for cancer classification, algorithmic opacity and absence of interpretability frameworks tailored to resource-constrained environments have impeded clinical adoption. This study bridges the translational gap between predictive accuracy and clinical utility by developing an explainable artificial intelligence (XAI) framework specifically designed for breast cancer diagnosis in underserved healthcare settings. Using the Wisconsin Breast Cancer Diagnostic Dataset (569 fine-needle aspirate cytological specimens with 30 nuclear morphometric features), we systematically benchmarked eight supervised classification algorithms: Logistic Regression, Random Forest, XGBoost, LightGBM, Support Vector Machine (SVM), Gradient Boosting, Decision Tree, and K-Nearest Neighbors, using stratified 10-fold cross-validation and an independent hold-out test set (80:20 split). Performance was evaluated across discriminative and probabilistic metrics, including AUC-ROC, F1-score, Matthews Correlation Coefficient (MCC), and Brier score, and interpretability was operationalized through SHapley Additive exPlanations (SHAP) analysis with global feature importance, cross-model consensus ranking, and individual-level dependence characterization. All ensemble and regularized models achieved test-set AUCs above 0.98, with XGBoost and SVM attaining the highest AUC of 0.996, and Logistic Regression the highest accuracy (98.25%) and MCC (0.962). SHAP analysis consistently identified worst perimeter, worst concave points, and worst area as the dominant predictors, with strong concordance across gradient-boosted models (pairwise Spearman rho: XGBoost–LightGBM 0.86, XGBoost–Random Forest 0.82, Random Forest–LightGBM 0.67). Logistic Regression also demonstrated superior probability calibration, a critical requirement for clinical risk stratification. Collectively, these findings deliver a reproducible, transparent framework whose SHAP-derived signatures align with established cytopathological principles, supporting responsible integration of interpretable ML into resource-limited diagnostic workflows and providing a template for equitable AI deployment in global oncology.

## Author summary

Breast cancer is the most commonly diagnosed cancer among women worldwide, yet survival rates remain dramatically lower in low- and middle-income countries (LMICs) compared to high-income nations, largely due to limited access to specialist diagnostic expertise. Machine learning (ML) holds promise for supporting cancer diagnosis in these settings, but most high-performing ML systems function as difficult-to-interpret ‘black boxes,’ undermining clinician trust and adoption, particularly in environments where algorithmic outputs cannot be readily verified by specialist pathologists. In this study, we developed and evaluated an interpretable ML framework for breast cancer classification using fine-needle aspiration cytology data routinely collected in resource-limited settings. By systematically comparing eight ML algorithms and applying SHAP (SHapley Additive exPlanations)-based explainability analysis, we identified which cellular features most strongly influence diagnostic predictions and demonstrated that these features align with established cytopathological criteria. Crucially, we show that simpler, more interpretable models achieve performance comparable to complex ensembles while offering superior probability calibration, a critical property for clinical triage decisions. Our findings support the use of transparent, computationally lightweight ML systems as viable decision-support tools in underserved healthcare environments, advancing the goal of equitable cancer diagnosis globally.

## 1. Introduction

Breast cancer constitutes the most prevalent malignancy and the leading cause of cancer-related mortality among women worldwide, with an estimated 2.3 million new cases and 685,000 deaths reported annually [1,2]. The global distribution of breast cancer burden, however, is markedly asymmetric: while five-year survival rates exceed 90% in high-income countries (HICs), they remain below 40%–60% in many low- and middle-income countries (LMICs) across Sub-Saharan Africa, South Asia, and parts of Latin America [3,4]. This disparity is principally attributable to diagnostic delays, limited access to histopathological expertise, inadequate screening infrastructure, and fragmented referral pathways that collectively impede early detection and timely therapeutic intervention [5,6].

Fine-needle aspiration cytology (FNAC) represents a cost-effective, minimally invasive diagnostic modality that is particularly well-suited to resource-constrained environments where core biopsy and immunohistochemistry capabilities may be unavailable [7]. The digitization of FNAC specimens and extraction of quantitative nuclear morphometric features has created opportunities for computational diagnostic support, yet the translation of these computational advances into routine clinical practice in LMICs remains profoundly limited [8,9].

Machine learning (ML) has emerged as a transformative paradigm in computational oncology, with numerous studies demonstrating classification accuracies exceeding 95% for breast lesion characterization [10,11]. However, the predominant focus on predictive performance metrics has obscured three critical translational barriers. First, many high-performing ML models function as algorithmic “black boxes”, generating predictions without clinically interpretable reasoning chains, thereby undermining clinician trust and adoption [12]. Second, the computational and infrastructural requirements of complex deep learning architectures often exceed the technological capacity of LMIC healthcare facilities [13]. Third, the absence of standardized explainability frameworks impedes regulatory approval and ethical deployment of ML-assisted diagnostics in contexts where algorithmic errors may carry disproportionate consequences due to limited recourse mechanisms [14,15].

Explainable artificial intelligence (XAI) has emerged as a principled response to these challenges. Among XAI methodologies, SHapley Additive exPlanations (SHAP), grounded in cooperative game theory, provides a mathematically rigorous framework for decomposing model predictions into individual feature contributions [16]. SHAP offers several distinctive advantages for clinical applications: local interpretability (explaining individual predictions), global feature importance rankings, model-agnostic applicability, and consistency guarantees that ensure faithful representation of model behavior [17]. While several prior studies have applied SHAP to breast cancer ML classifiers [18–20], these analyses have received limited attention in the specific context of resource-limited settings, where computational parsimony and algorithmic transparency carry distinct operational weight. Prior SHAP-based analyses of breast cancer ML models have predominantly focused on single-model interpretability without systematic cross-model consensus analysis, probability calibration evaluation, or explicit contextualization for LMIC clinical deployment, dimensions that are central to the present study [21,22].

The present study addresses this translational gap through three interconnected objectives. First, we conduct a rigorous multi-algorithmic benchmarking study comparing eight supervised learning classifiers for breast cancer diagnosis using publicly accessible cytomorphometric data. Second, we implement a comprehensive SHAP-based explainability pipeline that characterizes global feature importance, cross-model consensus, and individual feature-outcome relationships. Third, we contextualize these findings within a clinically actionable decision-support framework explicitly designed for integration into LMIC diagnostic workflows, where algorithmic transparency, computational parsimony, and biological plausibility are essential prerequisites for responsible deployment [23,24].

By integrating predictive accuracy with mechanistic transparency and decision-support utility, this study contributes to the emerging paradigm of “Translational Equity” in AI-assisted oncology, which posits that equitable cancer care requires not only access to advanced diagnostic technologies but also the interpretability infrastructure necessary for clinicians in underserved settings to critically evaluate, trust, and meaningfully integrate algorithmic recommendations into patient care pathways [25,26].

## 2. Materials and methods

### 2.1. Ethics statement

This study utilized a de-identified, publicly available dataset and did not involve direct interaction with human subjects. Institutional review board (IRB) approval was not required per institutional policy for analyses of publicly accessible, anonymized data. The WBCD contains no personally identifiable information, demographic variables, or protected health information. All specimens were de-identified at source prior to public release. The study was conducted in accordance with the principles of responsible AI research, including transparency of methods, reproducibility of results, and open dissemination of analytical tools.

### 2.2. Study design and analytical framework

This study employed a retrospective, cross-sectional analytical design utilizing a publicly available benchmark dataset. The analytical pipeline comprised four sequential phases: (i) data acquisition and preprocessing, (ii) multi-algorithmic model development with hyperparameter optimization, (iii) comprehensive performance evaluation using discriminative and probabilistic metrics, and (iv) post-hoc explainability analysis using SHAP. The study adheres to the Transparent Reporting of a multivariable prediction model for Individual Prognosis Or Diagnosis (TRIPOD) guidelines and follows TRIPOD-ML extensions for machine learning-based prediction studies. A completed TRIPOD checklist is provided as supplementary material. All analyses were conducted in Python 3.10, leveraging scikit-learn (v1.3), XGBoost (v2.0), LightGBM (v4.1), and SHAP (v0.43) libraries. The complete analytical codebase and all supplementary materials are publicly available via a dedicated GitHub repository (see Data Availability Statement) to ensure full computational reproducibility in accordance with FAIR (Findable, Accessible, Interoperable, Reusable) data principles.

### 2.3. Data source and characteristics

We utilized the Wisconsin Breast Cancer Diagnostic Dataset (WBCD), originally curated by Dr. William H. Wolberg at the University of Wisconsin Hospitals and publicly accessible through the UCI Machine Learning Repository and scikit-learn library [27]. The WBCD comprises 569 instances representing digitized fine-needle aspirate (FNA) specimens from breast masses, with 212 malignant (37.3%) and 357 benign (62.7%) cases. Each specimen is characterized by 30 real-valued nuclear morphometric features computed from digitized cell nucleus images. These features capture ten distinct morphological properties of cell nuclei, each reported as mean, standard error (SE), and “worst” (mean of the three largest nuclei) values: radius, texture, perimeter, area, smoothness, compactness, concavity, concave points, symmetry, and fractal dimension [27].

The selection of this dataset was deliberate and methodologically motivated. The WBCD derives from FNAC, the cytological technique most commonly employed in LMICs for breast mass evaluation. Its morphometric features correspond to quantifiable nuclear properties assessable through digital microscopy, which is increasingly available even in peripheral health facilities. The dataset has been extensively validated in the ML literature, enabling meaningful benchmarking, while its manageable dimensionality (30 features) supports interpretable modeling without requiring the computational infrastructure demanded by high-dimensional omics data or deep learning on raw imaging data.

### 2.4. Data preprocessing and partitioning

The dataset contained no missing values. All 30 continuous features were standardized using z-score normalization (zero mean, unit variance) via the StandardScaler transformation, fitted exclusively on training data to prevent information leakage. The dataset was partitioned into training (80%, *n* = 455) and hold-out test (20%, *n* = 114) subsets using stratified random sampling (random seed = 42) to preserve the original class distribution in both partitions. The 80:20 ratio was selected based on statistical considerations appropriate to this dataset size; the resulting training set provides sufficient observations to support stable parameter estimation across all eight classifiers and 30 features, while the hold-out set (42 malignant, 72 benign) provides adequate statistical power for reliable performance estimation. This ratio complements the primary internal validation strategy of stratified 10-fold cross-validation applied to the training partition.

Prior to any model development, we verified the comparability of the two partitions through two checks: (i) class distribution confirmation, the malignant-to-benign ratio was preserved at approximately 37:63 in both the training (37.1% malignant) and test (36.8% malignant) subsets through stratified sampling; and (ii) feature distribution equivalence, Mann–Whitney *U* testing across all 30 features between the training and hold-out subsets revealed no statistically significant distributional differences (all *p* > 0.05 after Bonferroni correction), confirming that the hold-out partition constitutes a representative and unbiased sample of the source data prior to classifier development.

### 2.5. Classification algorithms

Eight supervised learning algorithms representing distinct inductive biases and learning paradigms were selected for systematic comparison (S1 Table provides the complete hyperparameter specifications for all models):

Logistic Regression (LR): A maximum-likelihood linear classifier with L2 regularization (C = 1.0) and LBFGS optimizer, serving as a transparent baseline with inherent interpretability.

Random Forest (RF): A bagging-based ensemble of 300 decision trees with maximum depth of 10, minimum samples per split of 5, and minimum samples per leaf of 2, providing implicit feature importance through impurity-based measures.

Extreme Gradient Boosting (XGBoost): A regularized gradient boosting framework with 200 estimators, maximum depth of 5, learning rate of 0.05, and column and row subsampling at 80%, incorporating L1 and L2 regularization.

Light Gradient Boosting Machine (LightGBM): A histogram-based gradient boosting algorithm with 200 estimators, maximum depth of 5, learning rate of 0.05, and 31 leaves, optimized for computational efficiency.

Support Vector Machine (SVM): A kernel-based classifier employing the Radial Basis Function (RBF) kernel with regularization parameter C = 10 and automatic gamma scaling, with Platt scaling for probability estimation.

Gradient Boosting Classifier (GBC): A sequential ensemble of 200 shallow decision trees (maximum depth = 4) with learning rate of 0.05 and stochastic subsampling at 80%.

Decision Tree (DT): A single CART classifier with maximum depth of 5 and minimum samples per split of 5, serving as an interpretable but potentially high-variance baseline.

K-Nearest Neighbors (KNN): A distance-based classifier with 7 neighbors, distance-weighted voting, and Minkowski metric, representing a non-parametric instance-based approach.

Hyperparameter Configuration and Optimization: The hyperparameter configurations were determined through a two-stage approach. In stage one, initial parameter ranges were informed by published algorithm-specific benchmarks for structured tabular data and developer recommendations [28,29]. In stage two, these ranges were refined through systematic grid search conducted exclusively on the training partition (*n* = 455) using 5-fold stratified cross-validation with AUC-ROC as the primary optimization criterion. For boosting algorithms, the search space encompassed: n_estimators ∈ {100, 200, 300, 500}, max_depth ∈ {3, 5, 7, 10}, and learning_rate ∈ {0.01, 0.05, 0.10, 0.20}. The hold-out test set was strictly withheld from all hyperparameter selection procedures to prevent information leakage. Final configurations represent those maximizing cross-validated AUC while maintaining training-to-validation performance stability, as evidenced by the learning curve analysis (S1 Fig).

### 2.6. Model evaluation strategy

Model performance was assessed through a dual evaluation protocol. Internal validation employed stratified 10-fold cross-validation (CV) on the training partition, reporting mean and standard deviation for accuracy, F1-score, and AUC-ROC. Hold-out test set evaluation utilized the pre-specified 20% partition, with the following metrics computed: accuracy, precision (positive predictive value), recall (sensitivity), F1-score, area under the receiver operating characteristic curve (AUC-ROC), Matthews Correlation Coefficient (MCC), Brier score, log loss, and average precision (area under the precision-recall curve). The selection of multiple complementary metrics was intentional. In cancer diagnostics, sensitivity (recall) is prioritized to minimize false negatives (missed malignancies), while precision and specificity govern false-positive rates that drive unnecessary procedures. MCC provides a balanced assessment robust to class imbalance, while Brier score and calibration analysis evaluate the probabilistic reliability essential for clinical risk communication [30].

We explicitly note that the hold-out test set, while derived through a pre-specified, strictly separated partitioning procedure, shares the same source dataset (WBCD) as the training partition and therefore constitutes internal hold-out validation, not true external validation as defined by TRIPOD-ML criteria. True external validation, requiring evaluation on an independent cytological dataset from a separate cohort, institution, or geographic context, was not feasible within the scope of this study and is designated as a priority for future work (Section 4.6).

### 2.7. SHapley Additive exPlanations (SHAP) explainability analysis

Post-hoc model interpretability was operationalized using SHAP (SHapley Additive exPlanations), a unified framework grounded in Shapley values from cooperative game theory [16]. For tree-based models (XGBoost, Random Forest, LightGBM), the optimized TreeExplainer algorithm was employed, which computes exact Shapley values in polynomial time by exploiting the recursive partitioning structure of decision trees.

The SHAP analysis comprised four components: (i) Global importance ranking, computed as the mean absolute SHAP value across all test instances for each feature, quantifying the average magnitude of each feature’s contribution to model output. (ii) Cross-model consensus analysis, comparing SHAP-derived feature rankings across XGBoost, Random Forest, and LightGBM to identify algorithmically robust predictive features versus model-dependent predictors. (iii) SHAP dependence analysis, examining the functional relationship between individual feature values and their SHAP contributions for the six most influential features, with quadratic trend estimation to characterize nonlinear effects. (iv) Probability calibration assessment using calibration curves (reliability diagrams) to evaluate the correspondence between predicted probabilities and observed outcome frequencies.

### 2.8. Clinical contextualization for resource-limited settings

To bridge the computational-clinical translation gap, model selection criteria incorporated considerations beyond predictive accuracy: (i) computational parsimony, favoring algorithms deployable on standard computing hardware without GPU requirements; (ii) interpretability depth, prioritizing models amenable to feature-level explanation; (iii) calibration quality, essential for communicating diagnostic certainty in settings with limited specialist oversight; and (iv) biological plausibility, evaluating whether SHAP-identified features align with established cytopathological diagnostic criteria.

### 2.9. Statistical analysis

Differences in feature distributions between malignant and benign classes were assessed using the Mann–Whitney *U* test, with significance defined as *p* < 0.05 after Bonferroni correction. Cross-model feature ranking concordance was evaluated using Spearman rank correlation. All confidence intervals are reported at the 95% level.

## 3. Results

### 3.1. Dataset characteristics and feature distribution

All 15 top SHAP-ranked features demonstrated highly significant distributional differences between diagnostic classes (Mann–Whitney *U* test, all *p* < 0.001, Bonferroni correction; Table 1), supporting their discriminative potential. Notably, “worst” (extreme-value) features exhibited the largest between-group effect sizes; worst perimeter showed the most pronounced malignant-benign separation (*p* < 2.58 × 10^−80^), consistent with the cytopathological recognition that malignant nuclei exhibit marked size heterogeneity [31]. Detailed descriptive statistics are provided in Table 1.

**Table 1:** Descriptive statistics of the top 15 SHAP-ranked features by diagnostic class.

Feature	Malignant (Mean ± SD)	Benign (Mean ± SD)	*U*-statistic	*p*-value
worst perimeter	141.370 ± 29.457	87.006 ± 13.527	73826.0	2.58e−80
worst concave points	0.182 ± 0.046	0.074 ± 0.036	73164.0	1.86e−77
worst area	1422.286 ± 597.968	558.899 ± 163.601	73400.5	1.80e−78
worst texture	29.318 ± 5.435	23.515 ± 5.494	59384.0	6.52e−30
area error	72.672 ± 61.355	21.135 ± 8.843	70114.5	5.77e−65
mean concave points	0.088 ± 0.034	0.026 ± 0.016	72992.5	1.01e−76
worst radius	21.135 ± 4.284	13.380 ± 1.981	73447.0	1.14e−78
worst concavity	0.451 ± 0.182	0.166 ± 0.140	69732.5	1.76e−63
mean smoothness	0.103 ± 0.013	0.092 ± 0.013	54647.0	7.79e−19
worst smoothness	0.145 ± 0.022	0.125 ± 0.020	57070.0	3.64e−24
mean texture	21.605 ± 3.779	17.915 ± 3.995	58717.5	3.43e−28
mean area	978.376 ± 367.938	462.790 ± 134.287	71015.5	1.54e−68
worst symmetry	0.323 ± 0.075	0.270 ± 0.042	55774.5	3.15e−21
mean compactness	0.145 ± 0.054	0.080 ± 0.034	65374.5	8.95e−48
compactness error	0.032 ± 0.018	0.021 ± 0.016	55043.5	1.17e−19

Values are presented as mean ± standard deviation. Statistical significance was assessed using the Mann–Whitney *U* test with Bonferroni correction (*n* = 569). *A*e − *n* = *A* × 10^−n^.

### 3.2. Comparative model performance

**Fig 1:**
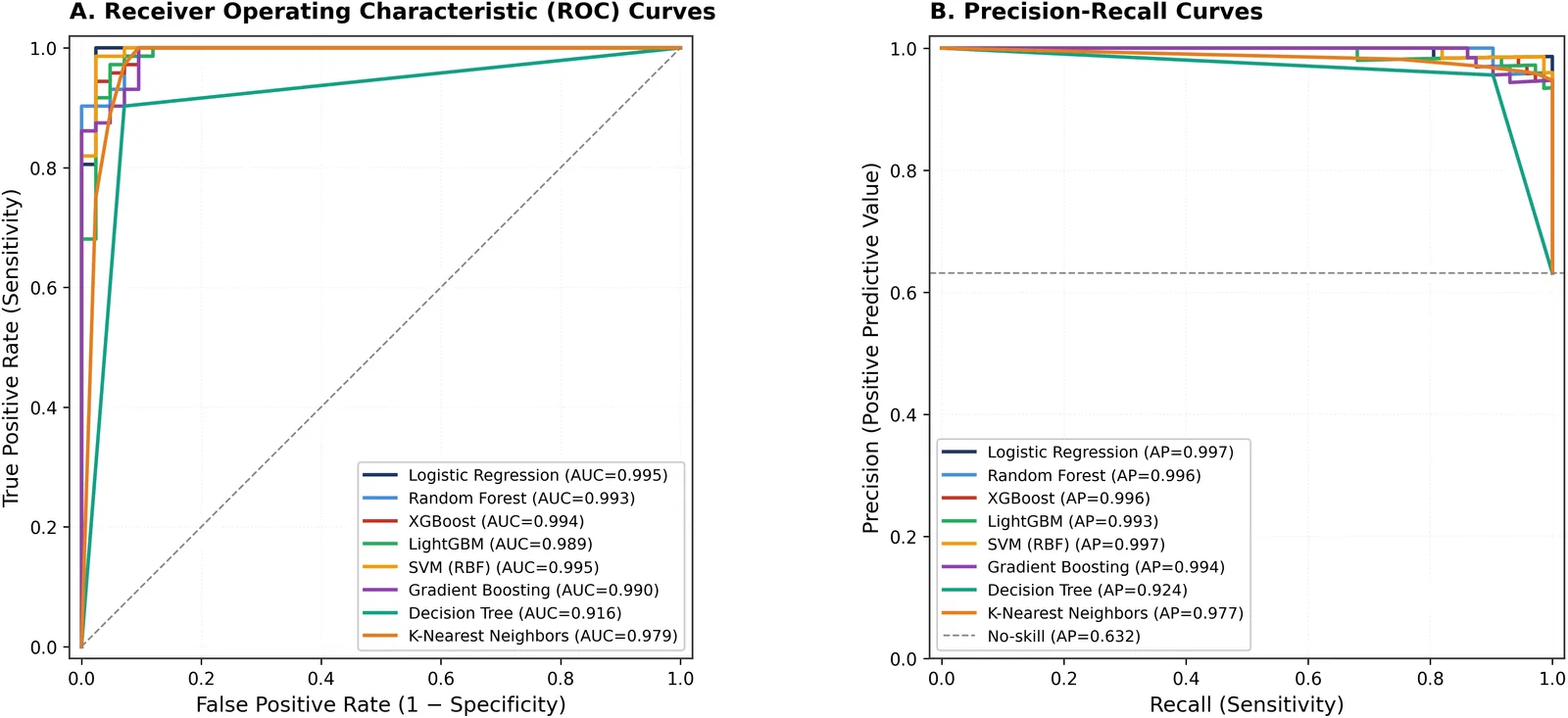
Receiver Operating Characteristic (ROC) and Precision-Recall (PR) curves for all eight classification algorithms evaluated on the independent test set (*n* = 114). **(A)** Representative ROC curves with area under the curve (AUC) values, and **(B)** precision-recall curves with average precision (AP) scores. The dashed diagonal line in Panel A represents random classifier performance (AUC = 0.5). All ensemble methods achieved AUC values exceeding 0.98, demonstrating near-ceiling discriminative performance.

On the hold-out test set (Table 2), AUC values ranged from 0.916 (Decision Tree) to 0.996 (XGBoost, SVM), with all ensemble methods exceeding 0.988 (Fig 1). Logistic Regression achieved the highest accuracy (0.983), precision (0.986), and MCC (0.962), while KNN attained perfect recall (1.000), the latter at the cost of three false positives, a trade-off requiring careful clinical consideration. The performance differential between the six top-performing models was narrow (accuracy: 0.956-0.983; AUC: 0.988-0.996), indicating that model selection should be guided primarily by interpretability and calibration rather than marginal accuracy differences. In 10-fold cross-validation, performance rankings were consistent with hold-out results (Table 2).

**Table 2:** Comparative performance of eight machine learning classifiers on the hold-out test set and stratified 10-fold cross-validation.

Model	Accuracy	Precision	Recall	F1-Score	AUC-ROC	MCC	Brier Score	CV Accuracy (10-fold)	CV AUC (10-fold)
Logistic Regression	0.983*	0.986*	0.986	0.986	0.995	0.962*	0.022	0.982 ± 0.013	0.995 ± 0.011
Random Forest	0.956	0.959	0.972	0.966	0.994	0.905	0.032	0.958 ± 0.034	0.988 ± 0.016
XGBoost	0.956	0.947	0.986	0.966	0.996*	0.906	0.029	0.971 ± 0.020	0.994 ± 0.010
LightGBM	0.965	0.960	0.986	0.973	0.989	0.925	0.031	0.969 ± 0.022	0.992 ± 0.012
SVM (RBF)	0.974	0.986	0.972	0.979	0.996*	0.944	0.026	0.972 ± 0.014	0.994 ± 0.013
Gradient Boosting	0.956	0.947	0.986	0.966	0.992	0.906	0.038	0.971 ± 0.028	0.992 ± 0.011
Decision Tree	0.921	0.957	0.917	0.936	0.916	0.834	0.070	0.936 ± 0.042	0.934 ± 0.053
K-Nearest Neighbors	0.974	0.960	1.000*	0.980	0.988	0.944	0.032	0.965 ± 0.026	0.989 ± 0.014

*Best values in each metric are bolded.

Abbreviations: AUC-ROC, Area Under the Receiver Operating Characteristic Curve; MCC, Matthews Correlation Coefficient; CV, Cross-Validation.

### 3.3. SHAP-based explainability analysis

#### 3.3.1. Global feature importance.

**Fig 2:**
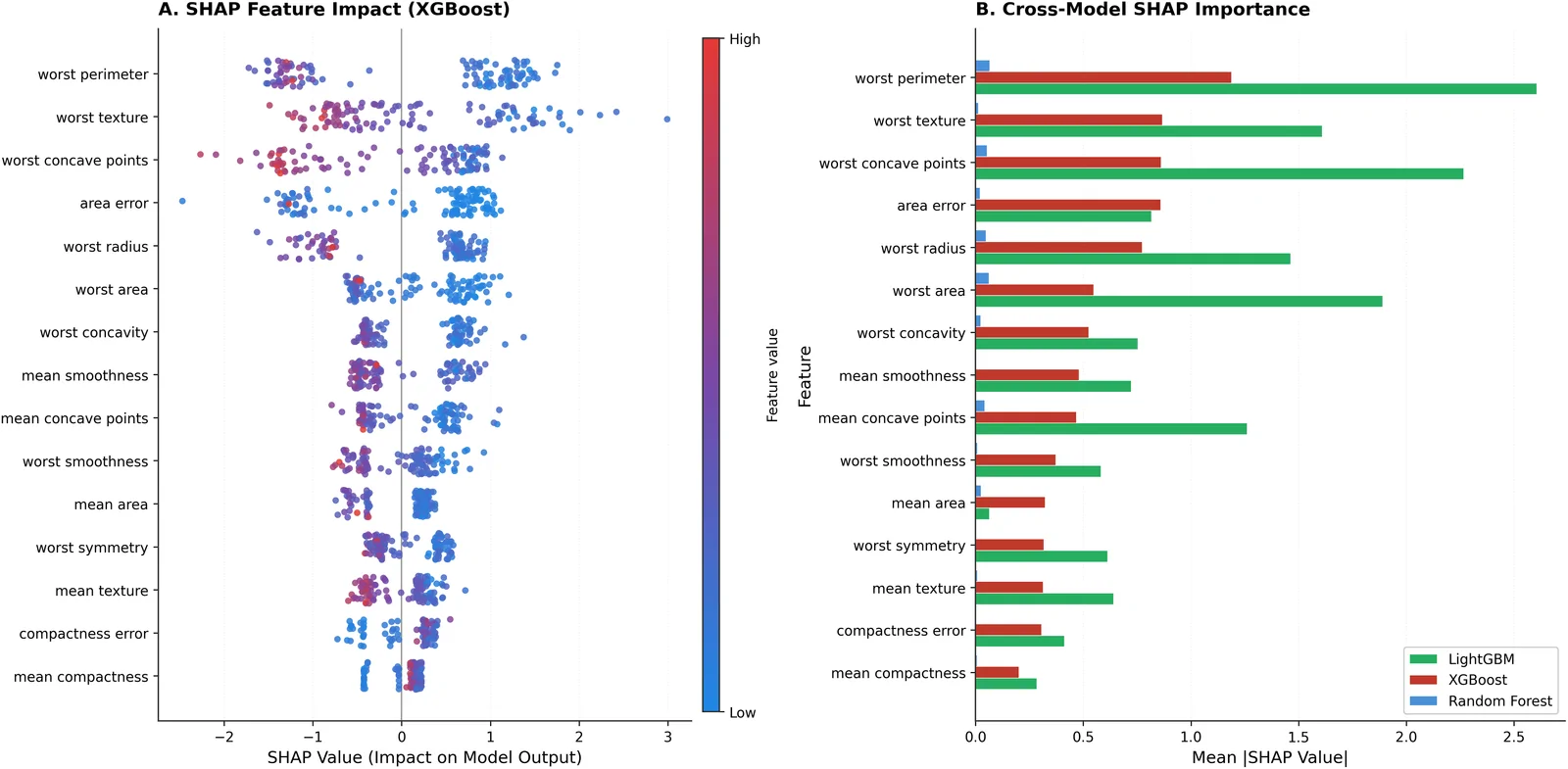
SHAP-based explainability analysis. **(A)** SHAP beeswarm plot for XGBoost showing the distribution of SHAP values for the top 15 features across all test instances. Each point represents one patient; color indicates the feature value (red = high, blue = low); horizontal position indicates the magnitude and direction of the feature’s impact on model output. **(B)** Comparative mean absolute SHAP values across XGBoost, Random Forest, and LightGBM, demonstrating cross-model feature importance consensus.

Worst perimeter (mean |SHAP| = 0.932), worst concave points (0.884), and worst area (0.778) were the three most important predictors in XGBoost (Fig 2A). Higher values of these features were associated with positive SHAP contributions (increased malignancy probability), consistent with cytopathological hallmarks of nuclear enlargement and irregular nuclear contours in malignant lesions [32].

#### 3.3.2. Cross-model SHAP consensus.

The three top-ranked features (worst perimeter, worst concave points, worst area) maintained consistent top-3 positions across all three models (Table 3; Fig 2B), with all three consensus metrics (arithmetic mean, geometric mean, and median rank) placing them identically. In contrast, worst texture showed pronounced model dependence (ranks: 4, 12, 6 across XGBoost/RF/LightGBM), and mean smoothness showed the greatest discordance (ranks: 9, 20, 10). Pairwise Spearman correlations of SHAP ranking vectors were XGBoost vs. LightGBM ρ = 0.86, XGBoost vs. RF *ρ* = 0.82, and RF vs. LightGBM *ρ* = 0.67, confirming that the two gradient-boosted architectures share greater feature importance concordance than either shares with the bagging-based RF.

**Table 3:** SHAP-derived feature importance rankings across three tree-based algorithms.

Feature	Mean |SHAP| (XGBoost)	XGBoost Rank	RF Rank	LightGBM Rank	Arithmetic Mean^*^	Geometric Mean†	Median^†^
**worst perimeter**	**0.932**	**1**	**2**	**2**	**1**	**1.6**	**2**
**worst concave points**	**0.884**	**2**	**3**	**1**	**2**	**1.8**	**2**
**worst area**	**0.778**	**3**	**1**	**3**	**2**	**2.1**	**3**
worst texture	0.743	4	12	6	7	6.6	6
area error	0.562	5	8	9	7	7.1	8
mean concave points	0.505	6	5	4	5	4.9	5
worst radius	0.436	7	4	5	5	5.2	5
worst concavity	0.347	8	10	7	8	8.2	8
mean smoothness	0.333	9	20	10	13	12.2	10
worst smoothness	0.299	10	15	11	12	11.8	11
mean texture	0.267	11	14	8	11	10.7	11
mean area	0.227	12	9	19	13	12.7	12
worst symmetry	0.201	13	19	12	14	14.4	13
mean compactness	0.151	14	17	15	15	15.3	15
compactness error	0.136	15	28	13	18	17.6	15
**Pairwise Spearman rank correlation coefficients between SHAP feature ranking vectors (*n* = 15 features shown):**							
		**XGBoost**		**Random Forest**		**LightGBM**	
**XGBoost**		–		0.82		0.86	
**Random Forest**		0.82		–		0.67	
**LightGBM**		0.86		0.67		–	

*Arithmetic Mean Rank = arithmetic mean of individual model ranks.

†Geometric Mean Rank and Median Rank are added to provide robustness against extreme values in the arithmetic mean. Shaded rows (top 3) demonstrate consistent top-tier importance across all three consensus metrics. Gradient-boosted architectures (XGBoost and LightGBM) demonstrate higher mutual SHAP ranking concordance (*ρ* = 0.86) than either shares with the bagging-based Random Forest.

### 3.4. Confusion matrix analysis

**Fig 3:**
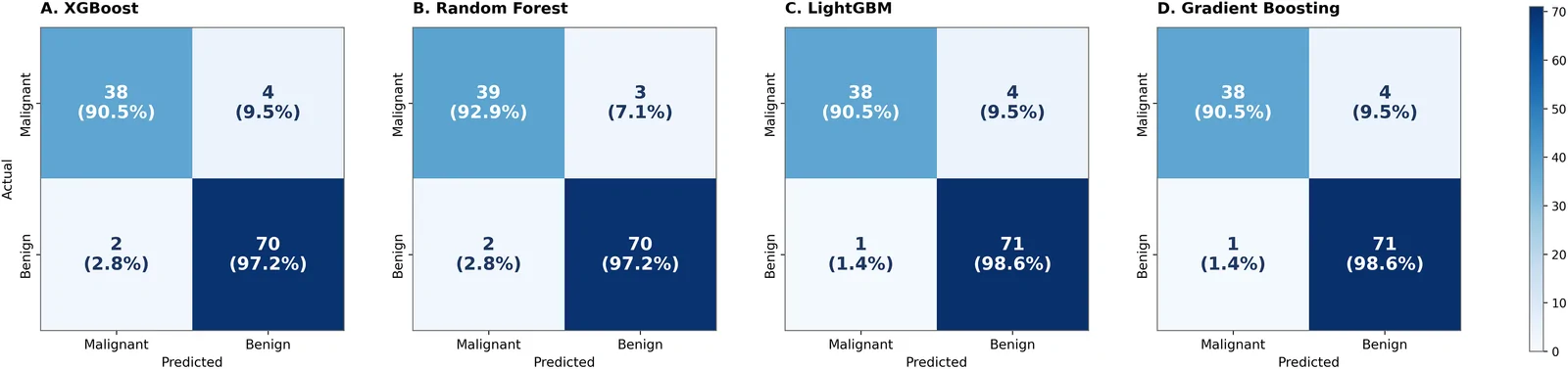
Confusion matrices for the four gradient boosting variants (A: XGBoost, B: Random Forest, C: LightGBM, D: Gradient Boosting) on the independent test set. Each cell displays the raw count and percentage of the true class. Darker shading indicates higher proportions. All models demonstrated strong true positive and true negative rates, with false negative counts (missed malignancies) ranging from 1 to 2.

Logistic Regression achieved the most favorable error profile for clinical deployment, with only 1 false negative (missed malignancy) and 1 false positive across 114 test cases (Fig 3). Full sensitivity, specificity, PPV, and NPV values for all eight models are provided in S2 Table.

### 3.5. Model calibration and multi-metric performance comparison

**Fig 4:**
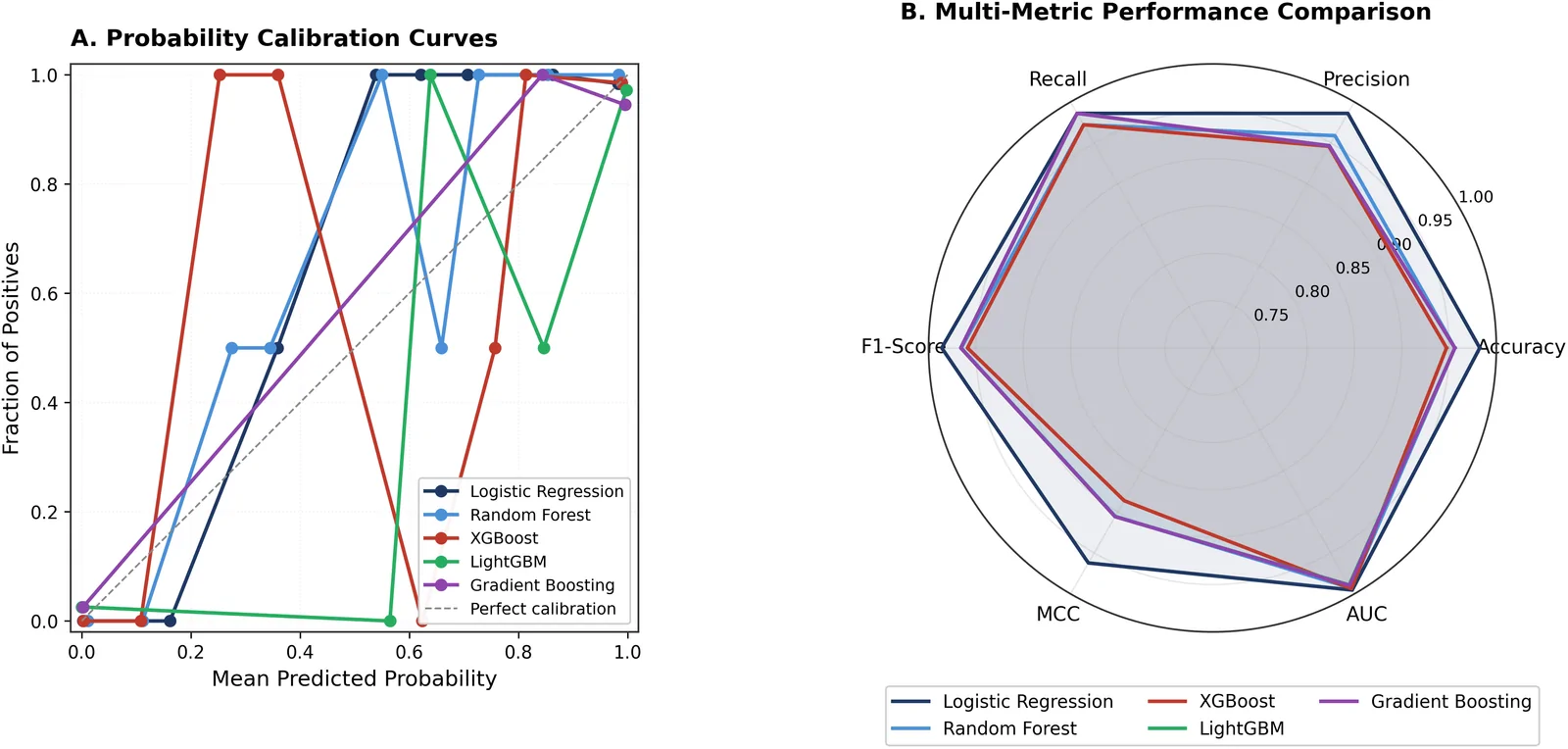
Model calibration and multi-metric performance comparison. **(A)** Probability calibration curves (reliability diagrams) for the five top-performing models. The dashed diagonal line represents perfect calibration; deviations indicate systematic over- or under-confidence in predicted probabilities. Logistic Regression demonstrated the best calibration fidelity. **(B)** Radar chart comparing six performance dimensions (Accuracy, Precision, Recall, F1-Score, AUC, normalized MCC) across the top five models.

Logistic Regression demonstrated the best calibration (Fig 4A), with predicted probabilities closely tracking observed frequencies. XGBoost showed modest mid-range overconfidence, and Random Forest exhibited characteristic sigmoidal miscalibration. The radar chart (Fig 4B) confirms that no single model dominates all six performance dimensions simultaneously, reinforcing context-specific selection guided by clinical priorities, Logistic Regression offering the most balanced overall profile.

### 3.6. SHAP dependence analysis

**Fig 5:**
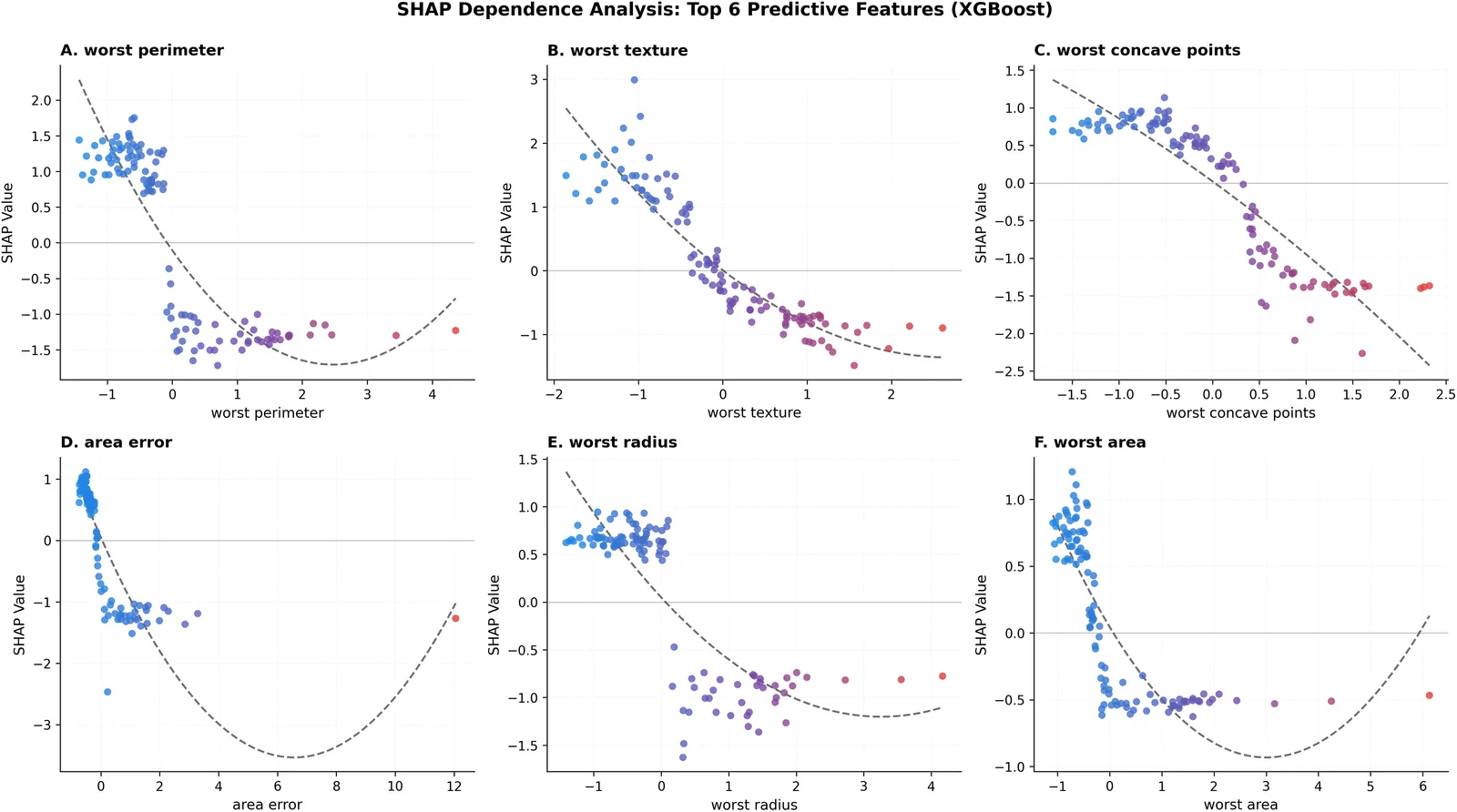
SHAP dependence plots for the six most influential predictive features identified by XGBoost. Each panel displays the relationship between individual feature values (x-axis) and their SHAP contributions to model output (y-axis). Points are colored by feature value magnitude (red = high, blue = low). Dashed curves represent quadratic trend fits. The horizontal gray line at SHAP = 0 delineates the transition between features pushing predictions toward benign (below) versus malignant (above) classification.

SHAP dependence plots (Fig 5) revealed that worst perimeter showed a strongly monotonic positive relationship consistent with a size threshold effect for malignancy, while worst concave points exhibited a steeper sigmoid-like profile reflecting its near-binary discriminative nature. Area error demonstrated a nonlinear relationship with heteroscedasticity at extreme values, potentially reflecting the cellular heterogeneity characteristic of malignant lesions.

## 4. Discussion

### 4.1. Principal findings and methodological contributions

This study demonstrates that a multi-algorithmic ML framework, augmented with SHAP-based explainability, achieves near-ceiling discriminative performance for FNAC-based breast cancer classification while providing transparent, clinically interpretable feature attributions. The principal methodological contribution of this study, relative to prior SHAP-based analyses of breast cancer classifiers, are fourfold: (i) the introduction of cross-model SHAP consensus analysis, using arithmetic mean, geometric mean, and median rank alongside pairwise Spearman correlations, as a method for distinguishing algorithmically invariant biological signals from model-specific artifacts; (ii) integration of probability calibration analysis as a deployment-critical evaluation dimension alongside discriminative metrics; (iii) systematic mapping of SHAP-derived feature importance to established cytopathological grading criteria (Masood Classification); and (iv) contextualization of all findings within the specific operational constraints and clinical priorities of LMIC diagnostic settings, a combination not previously addressed in the breast cancer SHAP literature [33,34].

While the WBCD has been extensively utilized in ML benchmarking studies, the vast majority of prior work has focused exclusively on maximizing classification accuracy without addressing clinical interpretability [33,35]. Key studies, including those by Agarap (2018) and Salod and Singh (2019), evaluated multiple classifiers but did not incorporate any explainability analysis, did not assess probability calibration, and made no attempt to contextualize their findings for clinical deployment in resource-limited settings. Our study advances beyond this body of work on the four dimensions outlined above. Our finding that Logistic Regression achieved the highest test accuracy (0.983) and MCC (0.962) while providing inherent interpretability calls into question the common assumption in computational oncology that model complexity is necessary for high diagnostic accuracy [35].

### 4.2. SHAP-derived clinical signatures: Biological plausibility and diagnostic relevance

The SHAP analysis identified worst perimeter, worst concave points, and worst area as the three most important predictive features across all tree-based models. These findings are consistent with the cytomorphological criteria codified in the Masood Classification System and other standardized FNAC grading schemas [36,37]. The dominance of “worst” features (mean of three largest nuclei) over “mean” features is biologically interpretable. Malignant tumors are characteristically heterogeneous, with subpopulations of cells exhibiting extreme morphological deviations. The “worst” features capture this intra-tumoral heterogeneity, effectively functioning as computational surrogates for the cytopathological practice of focusing on the most abnormal cells in a specimen [38]. This concordance between SHAP-identified feature importance and established diagnostic heuristics supports the biological plausibility and clinical relevance of the ML framework.

### 4.3. Implications for resource-limited settings and translational equity

We operationalize ‘Translational Equity’ as a three-component construct for evaluating AI-assisted diagnostics in resource-limited settings. The first component is technical accessibility: the AI system must be deployable within the computational and infrastructural constraints of the target setting, including standard hardware, offline functionality, and low inference latency. The second component is interpretive accessibility: the system must provide outputs that clinicians without specialist AI training can critically evaluate and meaningfully integrate, a requirement that calibrated probability outputs and SHAP-based explanations directly address. The third component is contextual alignment; the features identified as predictive must correspond to biological phenomena clinically recognizable and measurable within the diagnostic workflows of the target setting. A system that achieves high discriminative accuracy but fails on any one of these three components does not achieve Translational Equity, even on benchmark datasets. This distinction is critical because the primary bottleneck to equitable AI in global oncology is not the availability of algorithms but the absence of frameworks that make those algorithms trustworthy, usable, and contextually appropriate for the clinicians who most need them.

The translation of ML diagnostics to LMIC contexts requires careful consideration of several operational constraints that are frequently overlooked in algorithm development. First, computational infrastructure: all models evaluated in this study, including the ensemble methods, run efficiently on standard laptop hardware without GPU requirements, with inference times under 100 milliseconds per specimen, making them deployable on the computing resources available in peripheral health facilities [39].

Second, specialist scarcity: in many LMIC settings, a single cytopathologist may serve populations exceeding 500,000 [40]. An interpretable ML system that provides not only a diagnostic classification but also a transparent explanation of which morphometric features drove the prediction can serve as a “virtual second opinion,” potentially reducing inter-observer variability and supporting less-experienced technicians in preliminary screening. The SHAP-based explanation framework provides precisely this capability, highlighting for the user which specific cellular features were most influential in each individual prediction.

Third, calibration and risk communication: our calibration analysis demonstrates that Logistic Regression provides the most reliable probability estimates, which is critical for triage decisions in settings where confirmatory testing may require referral to distant facilities. A calibrated probability of 92% malignancy conveys meaningfully different clinical urgency than 60%, enabling more rational resource allocation in capacity-constrained environments [41].

Fourth, algorithmic accountability: the deployment of ML diagnostics in underserved settings carries heightened ethical obligations, as patients may have limited access to appeal mechanisms if algorithmic errors occur. SHAP-based explanations provide an audit trail that enables post-hoc review of individual predictions, supporting the principles of algorithmic accountability and the right to explanation that are increasingly codified in AI governance frameworks [42].

Honest acknowledgment of implementation barriers is essential to avoid creating unrealistic expectations. In truly resource-limited settings, the proposed three-stage pathway faces significant structural challenges beyond technical feasibility. First, retrospective validation (Stage i) requires access to locally annotated FNAC datasets, a resource absent in most LMIC cytopathology laboratories where digital archiving is rarely standardized. Second, integration into laboratory information management systems (Stage ii) presupposes electronic health record infrastructure that many peripheral facilities lack. Third, digitization of FNAC specimens requires calibrated digital microscopes or flatbed scanners, equipment often inaccessible at sub-district facilities. Fourth, regulatory pathways for AI-assisted diagnostics in most LMICs remain undefined, creating approval uncertainty. These challenges reinforce rather than diminish the importance of the Translational Equity framework: future development must engage implementation scientists, health systems specialists, and LMIC clinician communities from the earliest stages.

### 4.4. Cross-model SHAP consensus as a robustness indicator

A methodological contribution of this study is the systematic comparison of SHAP-derived feature rankings across three tree-based architectures. The high concordance of the top three features across XGBoost, Random Forest, and LightGBM, confirmed across arithmetic mean, geometric mean, and median consensus metrics, provides evidence that these features represent algorithmically invariant signals rather than artifacts of a particular model’s inductive bias. Conversely, the model-dependent variability observed for features such as worst texture and mean smoothness suggests that these features may interact with algorithmic assumptions (e.g., feature splitting criteria, regularization strategies) in model-specific ways, warranting cautious interpretation [28].

Pairwise Spearman correlation analysis revealed that XGBoost and LightGBM (both gradient-boosted architectures) demonstrated higher mutual concordance (*ρ* = 0.86) than either shared with Random Forest (XGBoost vs. RF: *ρ* = 0.82; RF vs. LightGBM: *ρ* = 0.67), suggesting that algorithmic architecture, specifically the boosting versus bagging paradigm, partially determines feature ranking concordance for this dataset. The geometric mean and median consensus ranks are fully consistent with arithmetic mean rankings for the top 5 features, while the divergence observed for lower-ranked features (e.g., compactness error: arithmetic rank 18 vs. geometric rank 17.6) reflects the sensitivity of the arithmetic mean to extreme RF rank values (rank 28), confirming the value of the robust consensus metrics.

This consensus-based approach to SHAP analysis offers a practical framework for clinical validation: features that maintain high importance across multiple algorithmic paradigms are more likely to reflect genuine biological signals and are therefore more appropriate targets for clinical decision rules.

### 4.5. Utility, accessibility, and open science commitment

The complete codebase, encompassing data preprocessing, model training, SHAP analysis, visualization generation, and calibration assessment, is publicly deposited in a GitHub repository (https://github.com/drbamodu/explainable-ml-breast-cancer-lmic) under open-source MIT license and archived with a persistent DOI on Zenodo (https://doi.org/10.5281/zenodo.18928723). All dependencies are version-pinned, and the analysis can be reproduced on standard computing hardware (no GPU required) within approximately 15 minutes, lowering the barrier to replication by researchers in resource-constrained institutions.

From a practical deployment perspective, the framework is designed to be modular and extensible. The trained Logistic Regression model, identified as the optimal candidate for LMIC deployment due to its combination of near-ceiling accuracy, superior calibration, inherent interpretability, and minimal computational requirements, can be exported as a serialized object and integrated into existing digital pathology workflows via a lightweight application programming interface (API). We envision a three-phase implementation pathway: (i) retrospective validation on locally collected FNAC datasets to assess domain generalizability; (ii) integration into laboratory information management systems (LIMS) as a computer-aided detection (CADx) module; and (iii) prospective evaluation in a clinical effectiveness trial comparing ML-augmented versus standard cytopathological assessment. The SHAP explanation layer can be rendered as a visual overlay highlighting which morphometric features contributed most to each individual prediction, enabling cytotechnicians and trainee pathologists to engage critically with algorithmic outputs rather than treating them as opaque directives.

With respect to reporting standards and responsible AI, this study adheres to the TRIPOD-ML guidelines for transparent reporting of prediction model studies. We acknowledge the broader ethical imperative that ML systems deployed in healthcare settings, particularly those serving vulnerable populations, must be subject to continuous monitoring for performance drift, demographic bias, and calibration degradation over time. While the WBCD does not contain demographic variables that would enable formal fairness auditing, we emphasize that prospective deployments of this framework must incorporate systematic bias assessment as a prerequisite for ethical implementation.

### 4.6. Limitations and future directions

Several limitations warrant acknowledgment. First, the WBCD, while well-validated and FNAC-derived, originates from a single institution in the United States, and its generalizability to cytological specimens processed in LMIC laboratories with potentially different preparation protocols, staining techniques, and digitization equipment remains to be established through prospective multi-site validation studies [43]. Second, the binary classification framework (malignant vs. benign) does not capture the diagnostic complexity of indeterminate or atypical cytological findings, which represent a significant proportion of FNAC specimens and pose the greatest diagnostic challenge [44]. Third, while SHAP provides post-hoc interpretability, it does not establish causal relationships between features and outcomes; the identified associations, though biologically plausible, require prospective mechanistic validation.

Fourth, a key limitation of this study is the absence of true external validation. The hold-out test set utilized for performance evaluation was derived from the same WBCD dataset as the training partition and therefore does not fulfill TRIPOD-ML criteria for external validation, which requires evaluation on an independent dataset from a separate cohort, institution, or geographic context. The performance estimates reflect internal generalizability to this dataset’s distributional properties rather than generalizability to cytological specimens from LMIC laboratories. External validation on independent FNAC datasets from LMIC facilities represents the single most critical methodological priority before any clinical deployment can be responsibly considered. We are currently pursuing collaborative partnerships with cytopathology laboratories in Sub-Saharan Africa and South Asia for prospective collection and analysis.

Fifth, while hyperparameter optimization was conducted through cross-validated grid search on the training partition, the bounded search space and single optimization criterion (AUC-ROC) may not have identified globally optimal configurations for all performance dimensions. For hyperparameter-sensitive algorithms such as XGBoost and SVM, more exhaustive strategies (e.g., Bayesian optimization) may yield marginal gains, though the near-ceiling performance observed suggests these would be clinically inconsequential for this dataset. Sixth, sensitivity analyses across alternative split ratios (e.g., 70:30, 75:25) were not formally conducted; however, the primary 10-fold cross-validation substantially mitigates split-ratio dependency concerns.

Future research should prioritize external validation using cytological datasets from LMIC facilities, extension to multi-class classification incorporating indeterminate categories, integration with digital pathology workflows through application programming interfaces (APIs), and prospective clinical trials evaluating the impact of SHAP-augmented ML on diagnostic accuracy and turnaround time in resource-limited settings. Additionally, the development of model-embedded explanations that provide real-time interpretability during clinical use, rather than post-hoc analysis, represents a critical frontier for clinical XAI [22].

## 5. Conclusions

This study presents a reproducible, interpretable ML framework for FNAC-based breast cancer classification that achieves strong predictive accuracy (AUC up to 0.996) while maintaining clinical transparency through comprehensive SHAP-based explainability. The framework identifies biologically plausible morphometric signatures, specifically worst perimeter, worst concave points, and worst area, that exhibit robust predictive importance across multiple algorithmic paradigms and are consistent with established cytopathological diagnostic criteria. Critically, we demonstrate that interpretable models (Logistic Regression) achieve performance comparable to complex ensembles while offering superior calibration and inherent explainability, a finding with profound implications for deployment in resource-constrained settings where algorithmic transparency and probabilistic reliability are prerequisites for clinician trust and responsible integration into patient care. This work contributes to the emerging paradigm of Translational Equity in AI-assisted oncology, demonstrating that equitable cancer diagnosis requires not merely accurate algorithms but transparent, interpretable, and contextually appropriate decision-support systems that empower clinicians across all resource levels to deliver evidence-based care.

## Supporting information

S1 Table Model hyperparameters employed for all eight classification algorithms. (DOCX)

S2 Table Detailed confusion matrix metrics for all models including true positives (TP), true negatives (TN), false positives (FP), false negatives (FN), sensitivity, specificity, positive predictive value (PPV), and negative predictive value (NPV). (DOCX)

S1 Fig Learning curves for six machine learning classifiers showing training accuracy (blue) and 5-fold cross-validation accuracy (red) as a function of training set size. Shaded regions represent ±1 standard deviation. Convergence of training and validation curves indicates absence of overfitting, while persistent gaps suggest potential for improvement with additional training data. XGBoost and LightGBM demonstrate the most favorable learning dynamics with rapid convergence and minimal generalization gap.
(TIF)

S2 Fig Pairwise Pearson correlation heatmap for the top 15 SHAP-ranked features. Lower-triangle display with correlation coefficients annotated. Strong positive correlations are observed among size-related features (worst perimeter, worst area, worst radius, mean area), reflecting the mathematical and biological interdependence of nuclear geometry. This multicollinearity pattern underscores the importance of SHAP-based importance over naive correlation-based feature selection, as SHAP accounts for feature interactions and redundancies within the model context.
(TIF)

## References

[1] Sung H, Ferlay J, Siegel RL. Global cancer statistics 2020: GLOBOCAN estimates of incidence and mortality worldwide for 36 cancers in 185 countries. CA Cancer J Clin. 2021;71(3):209–49. doi: 10.3322/caac.21660 33538338

[2] Bray F, Laversanne M, Sung H. Global cancer statistics 2022: GLOBOCAN estimates of incidence and mortality worldwide for 36 cancers in 185 countries. CA Cancer J Clin. 2024;74(3):229–63. doi: 10.3322/caac.21834 38572751

[3] Joko-Fru WY, Jedy-Agba E, Korir A, Ogunbiyi O, Dzamalala CP, Chokunonga E, et al. The evolving epidemic of breast cancer in sub-Saharan Africa: results from the African Cancer Registry Network. Int J Cancer. 2020;147(8):2131–41. doi: 10.1002/ijc.33014 32306390

[4] Ginsburg O, Bray F, Coleman MP, Vanderpuye V, Eniu A, Kotha SR, et al. The global burden of women’s cancers: a grand challenge in global health. Lancet. 2017;389(10071):847–60. doi: 10.1016/S0140-6736(16)31392-7 27814965 PMC6191029

[5] Brand NR, Qu LG, Chao A, Ilbawi AM. Delays and barriers to cancer care in low- and middle-income countries: a systematic review. Oncologist. 2019;24(12):e1371–80. doi: 10.1634/theoncologist.2019-0057 31387949 PMC6975966

[6] Chukmaitov AS, Kaidarova DR, Talaeyva ST, Sheppard VB, Xu H, Siangphoe U, et al. Analysis of delays in breast cancer treatment and late-stage diagnosis in Kazakhstan. Asian Pac J Cancer Prev. 2018;19(9):2519–25. doi: 10.22034/APJCP.2018.19.9.2519 30256046 PMC6249466

[7] Orell SR, Sterrett GF. Fine needle aspiration cytology. 5th ed. Elsevier; 2012.

[8] Xie J, Liu R, Luttrell J 4th, Zhang C. Deep learning based analysis of histopathological images of breast cancer. Front Genet. 2019;10:80. doi: 10.3389/fgene.2019.00080 30838023 PMC6390493

[9] Arevalo J, Cruz-Roa A, Arias V, Romero E, González FA. An unsupervised feature learning framework for basal cell carcinoma image analysis. Artif Intell Med. 2015;64(2):131–45. doi: 10.1016/j.artmed.2015.04.004 25976208

[10] Kourou K, Exarchos TP, Exarchos KP, Karamouzis MV, Fotiadis DI. Machine learning applications in cancer prognosis and prediction. Comput Struct Biotechnol J. 2014;13:8–17. doi: 10.1016/j.csbj.2014.11.005 25750696 PMC4348437

[11] Cruz JA, Wishart DS. Applications of machine learning in cancer prediction and prognosis. Cancer Inform. 2007;2:59–77. doi: 10.1177/117693510600200030 19458758 PMC2675494

[12] Rudin C. Stop explaining black box machine learning models for high stakes decisions and use interpretable models instead. Nat Mach Intell. 2019;1(5):206–15. doi: 10.1038/s42256-019-0048-x 35603010 PMC9122117

[13] Wahl B, Cossy-Gantner A, Germann S, Schwalbe NR. Artificial intelligence (AI) and global health: how can AI contribute to health in resource-poor settings?. BMJ Glob Health. 2018;3(4):e000798. doi: 10.1136/bmjgh-2018-000798 PMC6135465 30233828

[14] Amann J, Blasimme A, Vayena E, Frey D, Madai VI, Precise4Q consortium. Explainability for artificial intelligence in healthcare: a multidisciplinary perspective. BMC Med Inform Decis Mak. 2020;20(1):310. doi: 10.1186/s12911-020-01332-6 33256715 PMC7706019

[15] Char DS, Shah NH, Magnus D. Implementing machine learning in health care - addressing ethical challenges. N Engl J Med. 2018;378(11):981–3. doi: 10.1056/NEJMp1714229 29539284 PMC5962261

[16] Lundberg SM, Lee SI. A unified approach to interpreting model predictions. Adv Neural Inf Process Syst. 2017;30:4765–74.

[17] Lundberg SM, Erion G, Chen H, DeGrave A, Prutkin JM, Nair B, et al. From local explanations to global understanding with explainable ai for trees. Nat Mach Intell. 2020;2(1):56–67. doi: 10.1038/s42256-019-0138-9 32607472 PMC7326367

[18] Khater T, Hussain A, Bendardaf R, Talaat IM, Tawfik H, Ansari S, et al. An explainable artificial intelligence model for the classification of breast cancer. IEEE Access. 2025;13:5618–33. doi: 10.1109/access.2023.3308446

[19] Hussain SM, Buongiorno D, Altini N, Berloco F, Prencipe B, Moschetta M, et al. Shape-based breast lesion classification using digital tomosynthesis images: the role of explainable artificial intelligence. Appl Sci. 2022;12(12):6230. doi: 10.3390/app12126230

[20] Naji MA, Filali SE, Aarika K, Benlahmar EH, Abdelouhahid RA, Debauche O. Machine learning algorithms for breast cancer prediction and diagnosis. Proc Comput Sci. 2021;191:487–92. doi: 10.1016/j.procs.2021.07.062

[21] Tjoa E, Guan C. A Survey on explainable artificial intelligence (XAI): toward medical XAI. IEEE Trans Neural Netw Learn Syst. 2021;32(11):4793–813. doi: 10.1109/TNNLS.2020.3027314 33079674

[22] van der Velden BHM, Kuijf HJ, Gilhuijs KGA, Viergever MA. Explainable artificial intelligence (XAI) in deep learning-based medical image analysis. Med Image Anal. 2022;79:102470. doi: 10.1016/j.media.2022.102470 35576821

[23] Topol EJ. High-performance medicine: the convergence of human and artificial intelligence. Nat Med. 2019;25(1):44–56. doi: 10.1038/s41591-018-0300-7 30617339

[24] Rajkomar A, Dean J, Kohane I. Machine learning in medicine. N Engl J Med. 2019;380(14):1347–58. doi: 10.1056/NEJMra1814259 30943338

[25] Braveman P, Gottlieb L. The social determinants of health: it’s time to consider the causes of the causes. Public Health Rep. 2014;129 Suppl 2(Suppl 2):19–31. doi: 10.1177/00333549141291S206 24385661 PMC3863696

[26] Obermeyer Z, Powers B, Vogeli C, Mullainathan S. Dissecting racial bias in an algorithm used to manage the health of populations. Science. 2019;366(6464):447–53. doi: 10.1126/science.aax2342 31649194

[27] Wolberg WH, Street WN, Mangasarian OL. Machine learning techniques to diagnose breast cancer from fine-needle aspirates. Cancer Lett. 1995;77(2–3):163–71. doi: 10.1016/0304-3835(94)90099-x 8168063

[28] Chen T, Guestrin C. In: Proceedings of the 22nd ACM SIGKDD International Conference on Knowledge Discovery and Data Mining, 2016. 785–94. 10.1145/2939672.293978

[29] Ke G, Meng Q, Finley T, Wang T, Chen W, Ma W, Ye Q, Liu T-Y. LightGBM: a highly efficient gradient boosting decision tree. In: Guyon I, Luxburg UV, Bengio S, et al, editors. Advances in neural information processing systems 30 (NIPS 2017). Curran Associates; 2017:3146–54. Available from: http://papers.nips.cc/paper/6907-lightgbm-a-highly-efficient-gradient-boosting-decision-tree.pdf

[30] Steyerberg EW, Vickers AJ, Cook NR, Gerds T, Gonen M, Obuchowski N, et al. Assessing the performance of prediction models: a framework for traditional and novel measures. Epidemiology. 2010;21(1):128–38. doi: 10.1097/EDE.0b013e3181c30fb2 20010215 PMC3575184

[31] Elmore JG, Longton GM, Carney PA, Geller BM, Onega T, Tosteson ANA, et al. Diagnostic concordance among pathologists interpreting breast biopsy specimens. JAMA. 2015;313(11):1122–32. doi: 10.1001/jama.2015.1405 25781441 PMC4516388

[32] Fischer AH, Jacobson KA, Rose J, Zeller R. Hematoxylin and eosin staining of tissue and cell sections. CSH Protoc. 2008;2008:pdb.prot4986. doi: 10.1101/pdb.prot4986 21356829

[33] Agarap AF. On breast cancer detection: an application of machine learning algorithms on the Wisconsin diagnostic dataset. In: Proceedings of the 2nd International Conference on Machine Learning and Soft Computing. ACM; 2018:5–9. doi:10.48550/arXiv.1711.07831

[34] Chaurasia V, Pal S. A novel approach for breast cancer detection using data mining techniques. Int J Innov Res Comput Commun Eng. 2020;8(2):2456–61.

[35] Salod Z, Singh Y. Comparison of the performance of machine learning algorithms in breast cancer screening and detection: a protocol. J Public Health Res. 2019;8(3):1677. doi: 10.4081/jphr.2019.1677 31857990 PMC6902303

[36] Masood S. Cytomorphology of fibrocystic change, high-risk proliferative breast disease, and premalignant breast lesions. Clin Lab Med. 2005;25(4):713–31, vi. doi: 10.1016/j.cll.2005.08.005 16308088

[37] Ali HR, Dariush A, Provenzano E, Bardwell H, Abraham JE, Iddawela M, et al. Computational pathology of pre-treatment biopsies identifies lymphocyte density as a predictor of response to neoadjuvant chemotherapy in breast cancer. Breast Cancer Res. 2016;18(1):21. doi: 10.1186/s13058-016-0682-8 26882907 PMC4755003

[38] Marusyk A, Almendro V, Polyak K. Intra-tumour heterogeneity: a looking glass for cancer?. Nat Rev Cancer. 2012;12(5):323–34. doi: 10.1038/nrc3261 22513401

[39] Hosny A, Parmar C, Quackenbush J, Schwartz LH, Aerts HJWL. Artificial intelligence in radiology. Nat Rev Cancer. 2018;18(8):500–10. doi: 10.1038/s41568-018-0016-5 29777175 PMC6268174

[40] Jedy-Agba E, McCormack V, Adebamowo C, dos-Santos-Silva I. Stage at diagnosis of breast cancer in sub-Saharan Africa: a systematic review and meta-analysis. Lancet Glob Health. 2016;4(12):e923–35. doi: 10.1016/S2214-109X(16)30259-5 PMC5708541 27855871

[41] Van Calster B, McLernon DJ, van Smeden M, Wynants L, Steyerberg EW, Topic Group ‘Evaluating diagnostic tests and prediction models’ of the STRATOS initiative. Calibration: the Achilles heel of predictive analytics. BMC Med. 2019;17(1):230. doi: 10.1186/s12916-019-1466-7 31842878 PMC6912996

[42] Vayena E, Blasimme A, Cohen IG. Machine learning in medicine: addressing ethical challenges. PLoS Med. 2018;15(11):e1002689. doi: 10.1371/journal.pmed.1002689 30399149 PMC6219763

[43] Johnson AEW, Pollard TJ, Shen L, Lehman L-WH, Feng M, Ghassemi M, et al. MIMIC-III, a freely accessible critical care database. Sci Data. 2016;3:160035. doi: 10.1038/sdata.2016.35 27219127 PMC4878278

[44] Nigam JS, Kumar T, Bharti S, Surabhi, Sinha R, Bhadani PP. The International Academy of Cytology standardized reporting of breast fine-needle aspiration biopsy cytology: a 2 year’s retrospective study with application of categories and their assessment for risk of malignancy. Cytojournal. 2021;18:27. doi: 10.25259/Cytojournal_43_2020 34876918 PMC8645495

