# Supplementary material for: Explainable machine learning for breast cancer prediction in resource-constrained settings: A multi-algorithmic framework integrating shap-based transparency with clinical decision support
Source: PLOS Digit Health. 2026 Sep 11;5(9):e0001706. doi: 10.1371/journal.pdig.0001706 (PMC13568489; doi:10.1371/journal.pdig.0001706)
Supplement: Supplementary file 1 [file pdig.0001706.s001.docx]

**S1 Table.** Model hyperparameters employed for all eight classification algorithms.

| **Model** | **Key Hyperparameters** |
| --- | --- |
| Logistic Regression | C=1.0, penalty=L2, solver=LBFGS |
| Random Forest | n_estimators=300, max_depth=10, min_samples_split=5, min_samples_leaf=2 |
| XGBoost | n_estimators=200, max_depth=5, learning_rate=0.05, subsample=0.8, colsample_bytree=0.8 |
| LightGBM | n_estimators=200, max_depth=5, learning_rate=0.05, num_leaves=31 |
| SVM (RBF) | C=10, gamma=scale, kernel=RBF |
| Gradient Boosting | n_estimators=200, max_depth=4, learning_rate=0.05, subsample=0.8 |
| Decision Tree | max_depth=5, min_samples_split=5 |
| K-Nearest Neighbors | n_neighbors=7, weights=distance, metric=minkowski |

All models were trained with a fixed random seed (42) to ensure reproducibility.
