# Supplementary material for: Explainable machine learning for breast cancer prediction in resource-constrained settings: A multi-algorithmic framework integrating shap-based transparency with clinical decision support
Source: PLOS Digit Health. 2026 Sep 11;5(9):e0001706. doi: 10.1371/journal.pdig.0001706 (PMC13568489; doi:10.1371/journal.pdig.0001706)
Supplement: Supplementary file 2 [file pdig.0001706.s002.docx]

**S2 Table.** Detailed confusion matrix metrics for all models, including true positives (TP), true negatives (TN), false positives (FP), false negatives (FN), sensitivity, specificity, positive predictive value (PPV), and negative predictive value (NPV).

| **Model** | **TP** | **TN** | **FP** | **FN** | **Sensitivity** | **Specificity** | **PPV** | **NPV** |
| --- | --- | --- | --- | --- | --- | --- | --- | --- |
| Logistic Regression | 71 | 41 | 1 | 1 | 0.9861 | 0.9762 | 0.9861 | 0.9762 |
| Random Forest | 70 | 39 | 3 | 2 | 0.9722 | 0.9286 | 0.9589 | 0.9512 |
| XGBoost | 71 | 38 | 4 | 1 | 0.9861 | 0.9048 | 0.9467 | 0.9744 |
| LightGBM | 71 | 39 | 3 | 1 | 0.9861 | 0.9286 | 0.9595 | 0.9750 |
| SVM (RBF) | 70 | 41 | 1 | 2 | 0.9722 | 0.9762 | 0.9859 | 0.9535 |
| Gradient Boosting | 71 | 38 | 4 | 1 | 0.9861 | 0.9048 | 0.9467 | 0.9744 |
| Decision Tree | 66 | 39 | 3 | 6 | 0.9167 | 0.9286 | 0.9565 | 0.8667 |
| K-Nearest Neighbors | 72 | 39 | 3 | 0 | 1.0000 | 0.9286 | 0.9600 | 1.0000 |
