# Supplementary material for: Explainable machine learning for breast cancer prediction in resource-constrained settings: A multi-algorithmic framework integrating shap-based transparency with clinical decision support
Source: PLOS Digit Health. 2026 Sep 11;5(9):e0001706. doi: 10.1371/journal.pdig.0001706 (PMC13568489; doi:10.1371/journal.pdig.0001706)
Supplement: Supplementary file 3 [file pdig.0001706.s006.docx]

**RESPONSE TO REVIEWERS**

Manuscript ID: PDIG-D-26-00399

Explainable Machine Learning for Breast Cancer Prediction in Resource-Constrained Settings:

A Multi-Algorithmic Framework Integrating SHAP-Based Transparency with Clinical Decision Support

We sincerely thank the Section Editor (Dr. Amara Tariq), Academic Editor (Prof. Jia-Lang Xu), Editor-in-Chief (Prof. Leo Anthony Celi), and both anonymous Reviewers for their thorough, constructive, and collegial evaluation of our manuscript. The reviewers' comments have substantially strengthened the scientific rigor, methodological transparency, and conceptual clarity of this work. We have addressed every comment in full, and the manuscript has been revised accordingly. Below, we provide a comprehensive point-by-point response.

For clarity, each reviewer comment is reproduced verbatim in a shaded box, followed by our detailed response and the specific revisions made to the manuscript. All revisions are identifiable in the submitted tracked-changes version. Page and section numbers refer to the revised manuscript.

**Managing Editor / Journal Requirements**

**Comment 1:**  We ask that a manuscript source file is provided at Revision. Please upload your manuscript file as a .doc, .docx, .rtf or .tex.

**Response 1:** As required, we have submitted our manuscript as a fully editable .docx file, which is uploaded as 'Manuscript' alongside this response. The tracked-changes version is uploaded as 'Revised Manuscript with Track Changes'.

**Comment 2:** Please upload separate figure files in .tif or .eps format. Also, remove the figures from your manuscript file but keep the legends.

**Response 2:** All five main figures and two supplementary figures have been extracted from the manuscript and are resubmitted as separate high-resolution .tif files processed through the PLOS NAAS tool. Figure legends are retained in the manuscript body at appropriate locations. The manuscript file no longer embeds the figures themselves.

**Comment 3:** Please provide an Author Summary. This should appear in your manuscript between the Abstract (if applicable) and the Introduction, and should be 150–200 words long. The aim should be to make your findings accessible to a wide audience that includes both scientists and non-scientists.

**Response 3:** A ~178-word Author Summary has been added to the manuscript between the Abstract and Introduction, as required. The summary is written to be accessible to both specialist and non-specialist readers, in accordance with PLOS Digital Health guidelines. Please see our newly added Author Summary section, page 4:

**Author Summary**

Breast cancer is the most commonly diagnosed cancer among women worldwide, yet survival rates remain dramatically lower in low- and middle-income countries (LMICs) compared to high-income nations, largely due to limited access to specialist diagnostic expertise. Machine learning (ML) holds promise for supporting cancer diagnosis in these settings, but most high-performing ML systems function as difficult-to-interpret 'black boxes,' undermining clinician trust and adoption, particularly in environments where algorithmic outputs cannot be readily verified by specialist pathologists. In this study, we developed and evaluated an interpretable ML framework for breast cancer classification using cytological data routinely collected in resource-limited settings. By systematically comparing eight ML algorithms and applying SHAP (SHapley Additive exPlanations)-based explainability analysis, we identified which cellular features most strongly influence diagnostic predictions and demonstrated that these features align precisely with established cytopathological criteria. Crucially, we show that simpler, more interpretable models achieve performance comparable to complex ensembles while offering superior probability calibration, a critical property for clinical triage decisions. Our findings support the use of transparent, computationally lightweight ML systems as viable decision-support tools in underserved healthcare environments, advancing the goal of equitable cancer diagnosis globally.

**Comment 4:** We notice that your supplementary tables are included in the manuscript file. Please remove them and upload them with the file type 'Supporting Information'. Please ensure that each Supporting Information file has a legend listed in the manuscript after the references list.

If the reviewer comments include a recommendation to cite specific previously published works, please review and evaluate these publications to determine whether they are relevant and should be cited. There is no requirement to cite these works unless the editor has indicated otherwise.

**Response 4:** Supplementary Tables S1 and S2, and Supplementary Figures S1 and S2 have been removed from the main manuscript body and resubmitted as individual Supporting Information files (S1_Table.docx, S2_Table.docx, S1_Fig.tif, S2_Fig.tif). Corresponding legends for each supporting information item are now listed in the manuscript after the References list, as required.

**Response to Reviewer #1 Comments**

We sincerely thank Reviewer 1 for their positive appraisal of the manuscript and their methodologically rigorous, specific critique. Each point has been addressed in full as detailed below.

**Comment 1A:** The authors present fixed hyperparameters for all eight classifiers (Supplementary Table S1) without describing any systematic optimization procedure. It is unclear whether these configurations were obtained through grid search, Bayesian optimization, randomized search, or manual tuning. This omission is consequential because improper hyperparameter selection can result in optimistically biased performance estimates. The authors should describe the hyperparameter selection strategy, including the validation method used.

**Response 1A:** We thank Reviewer 1 for this important methodological observation. This is a valid and consequential critique. We acknowledge that the absence of a clearly described hyperparameter optimization rationale represents a significant reporting gap. The hyperparameters reported in Supplementary Table S1 were not arbitrarily assigned; they were derived through a structured two-stage approach: (1) initial parameter ranges were informed by published algorithm-specific benchmarks and developer recommendations for structured tabular data; and (2) these ranges were refined through systematic grid search on the training partition using 5-fold cross-validation with AUC-ROC as the optimization criterion. For ensemble methods, the grid search encompassed n_estimators (100-500), max_depth (3-10), and learning_rate (0.01-0.20) for boosting algorithms. We have now fully described this hyperparameter optimization procedure in Section 2.4 of the revised manuscript. We have also explicitly acknowledged in Section 4.6 (Limitations) that the search space was bounded and that more exhaustive strategies (e.g., Bayesian optimization) may yield marginal additional gains, though the near-ceiling performance observed across models suggests these would be minimal for this dataset.

Please see our revised Methods 2.4, last paragraph:

Hyperparameter Configuration and Optimization: The hyperparameter configurations were determined through a two-stage approach. In stage one, initial parameter ranges were informed by published algorithm-specific benchmarks for structured tabular data and developer recommendations [28,29]. In stage two, these ranges were refined through systematic grid search conducted exclusively on the training partition (n = 455) using 5-fold stratified cross-validation with AUC-ROC as the primary optimization criterion. For boosting algorithms, the search space encompassed: n_estimators ∈ {100, 200, 300, 500}, max_depth ∈ {3, 5, 7, 10}, and learning_rate ∈ {0.01, 0.05, 0.10, 0.20}. The hold-out test set was strictly withheld from all hyperparameter selection procedures to prevent information leakage. Final configurations represent those maximizing cross-validated AUC while maintaining training-to-validation performance stability, as evidenced by the learning curve analysis (Supplementary Figure S1).

Also kindly see our revised Limitations 4.6:

Fourth, a key limitation of this study is the absence of true external validation. The hold-out test set utilized for performance evaluation was derived from the same WBCD dataset as the training partition and therefore does not fulfill TRIPOD-ML criteria for external validation, which requires evaluation on an independent dataset from a separate cohort, institution, or geographic context. The performance estimates reflect internal generalizability to this dataset's distributional properties rather than generalizability to cytological specimens from LMIC laboratories. External validation on independent FNAC datasets from LMIC facilities represents the single most critical methodological priority before any clinical deployment can be responsibly considered. We are currently pursuing collaborative partnerships with cytopathology laboratories in Sub-Saharan Africa and South Asia for prospective collection and analysis.

Fifth, while hyperparameter optimization was conducted through cross-validated grid search on the training partition, the bounded search space and single optimization criterion (AUC-ROC) may not have identified globally optimal configurations for all performance dimensions. For hyperparameter-sensitive algorithms such as XGBoost and SVM, more exhaustive strategies (e.g., Bayesian optimization) may yield marginal gains, though the near-ceiling performance observed suggests these would be clinically inconsequential for this dataset. Sixth, sensitivity analyses across alternative split ratios (e.g., 70:30, 75:25) were not formally conducted; however, the primary 10-fold cross-validation substantially mitigates split-ratio dependency concerns.

**Comment 1B:** The authors used an 80:20 train-test split, which is common; however, the manuscript does not justify its selection given the size of the dataset. The authors should briefly justify the choice of this split ratio and indicate whether alternative partitioning strategies were considered, and conduct sensitivity analyses across different split ratios.

**Response 1B:** We thank Reviewer 1 for this methodologically important observation. The 80:20 split was selected deliberately based on established statistical reasoning appropriate to the dataset size (n = 569). We have added a formal justification in Section 2.3, including the following considerations: (1) the training set size of n = 455 provides sufficient examples to support stable estimation across 30 features for all eight classifiers, including those with the highest variance (ensemble methods); (2) the hold-out set of n = 114 (including ~42 malignant and ~72 benign cases) provides adequate statistical power for performance estimation with meaningful confidence intervals; and (3) the selection complements the primary internal validation strategy of stratified 10-fold cross-validation, which directly addresses the sample-size concern by using 90% of training data iteratively. We have also confirmed distributional equivalence between partitions using Mann-Whitney U testing across all 30 features. Regarding sensitivity analysis across alternative split ratios (e.g., 70:30, 75:25): we acknowledge this as a valuable robustness check and have added it to the Limitations section with a note that the primary internal validation via 10-fold CV largely mitigates concerns about split-ratio dependency.

Please kindly see revised Methods 2.3, added after split description:

The 80:20 ratio was selected based on statistical considerations appropriate to this dataset size; the resulting training set provides sufficient observations to support stable parameter estimation across all eight classifiers and 30 features, while the hold-out set (42 malignant, 72 benign) provides adequate statistical power for reliable performance estimation. This ratio complements the primary internal validation strategy of stratified 10-fold cross-validation applied to the training partition.

Prior to any model development, we verified the comparability of the two partitions through two checks: (i) class distribution confirmation, the malignant-to-benign ratio was preserved at approximately 37:63 in both the training (37.1% malignant) and test (36.8% malignant) subsets through stratified sampling; and (ii) feature distribution equivalence, Mann-Whitney U testing across all 30 features between the training and hold-out subsets revealed no statistically significant distributional differences (all p > 0.05 after Bonferroni correction), confirming that the hold-out partition constitutes a representative and unbiased sample of the source data prior to classifier development.

Also see our revised Limitations 4.6:

Sixth, sensitivity analyses across alternative split ratios (e.g., 70:30, 75:25) were not formally conducted; however, the primary 10-fold cross-validation substantially mitigates split-ratio dependency concerns.

**Comment 1C:** The manuscript refers to the 20% hold-out split dataset as 'external validation.' However, if both the training and test sets were derived from the same original dataset, this does not constitute true external validation. External validation requires evaluation on an independent dataset obtained from a different cohort, institution, or source. The absence of genuine external validation limits confidence in the model's generalizability and real-world applicability. The authors should include an evaluation on an independent external dataset.

**Response 1C:** Reviewer 1 is entirely correct, and we are grateful for this important terminological and methodological correction. The 20% hold-out partition, derived from the same WBCD dataset as the training set, constitutes internal hold-out validation, not external validation in the rigorous TRIPOD sense, which requires an independent dataset from a separate cohort or institution. We have systematically corrected this terminology throughout the revised manuscript (Methods 2.5, Results 3.2, and Discussion 4.6), replacing all instances of 'external validation' with 'hold-out test set evaluation' or 'internal hold-out validation' as appropriate. We have also substantially expanded Section 4.6 to address this limitation candidly and prominently, acknowledging that true external validation, particularly on cytological datasets from LMIC facilities with different preparation protocols and imaging equipment, is the critical next methodological step before any clinical deployment can be responsibly considered. Regarding the request to include evaluation on an independent external dataset: we genuinely share the reviewer's view that true external validation is essential. However, no publicly available cytological FNAC dataset with comparable feature extraction pipelines and LMIC provenance currently exists that would constitute a valid external validation cohort for the WBCD-trained models. We have explicitly stated this constraint in Section 4.6 and designated it as the primary direction for future research, including prospective multi-site validation through partnerships with LMIC cytology laboratories. We note that, consistent with TRIPOD-ML guidance for development studies using public benchmark datasets, the disclosure of this limitation rather than artificially inflated generalizability claims represents the responsible reporting approach.

Please see our revised Methods 2.5, where terminology has been corrected throughout]:

All instances of 'external validation' referencing the 20% hold-out set have been replaced with 'hold-out test set evaluation' or 'internal hold-out validation.' The following clarifying statement has been added:

We explicitly note that the hold-out test set, while derived through a pre-specified, strictly separated partitioning procedure, shares the same source dataset (WBCD) as the training partition and therefore constitutes internal hold-out validation, not true external validation as defined by TRIPOD-ML criteria. True external validation, requiring evaluation on an independent cytological dataset from a separate cohort, institution, or geographic context, was not feasible within the scope of this study and is designated as the primary direction for future research (see Section 4.6).

Also see our substantial expansion of Limitations 4.6:

Fourth, a key limitation of this study is the absence of true external validation. The hold-out test set utilized for performance evaluation was derived from the same WBCD dataset as the training partition and therefore does not fulfill TRIPOD-ML criteria for external validation, which requires evaluation on an independent dataset from a separate cohort, institution, or geographic context. The performance estimates reflect internal generalizability to this dataset's distributional properties rather than generalizability to cytological specimens from LMIC laboratories. External validation on independent FNAC datasets from LMIC facilities represents the single most critical methodological priority before any clinical deployment can be responsibly considered. We are currently pursuing collaborative partnerships with cytopathology laboratories in Sub-Saharan Africa and South Asia for prospective collection and analysis.

**Comment 1D:** Can the authors confirm how they checked for the similarity between the training set and the hold-out dataset before the development of the classifier?

**Response 1D:** We thank Reviewer 1 for this important process question. Two checks were performed prior to any model development to confirm the comparability of the training and hold-out partitions: (1) Class distribution verification, stratified random sampling (random seed = 42) was used to ensure proportional representation of the malignant and benign classes in both partitions (training: 37.1% malignant, 62.9% benign; test: 36.8% malignant, 63.2% benign), confirming distributional equivalence for the target variable. (2) Feature distribution comparability, the Mann-Whitney U test was applied to compare the distribution of each of the 30 features between training and hold-out subsets. No statistically significant differences were detected for any feature (all p > 0.05 after Bonferroni correction across 30 comparisons), confirming that the hold-out partition constitutes a representative and unbiased sample of the source data. These checks are now explicitly reported in Section 2.3 of the revised manuscript (see also Response to Comment 1B above).

**Response to Reviewer #2 Comments**

We thank Reviewer 2 for their detailed and constructive review, which has significantly improved the precision, rigor, and presentation of the manuscript.

**Comment 1:** It is recommended to clearly state the specific differences from existing SHAP research in the introduction and discussion sections, avoiding excessive claims of being first-time or blank.

**Response 1:** We thank Reviewer 2 for this nuanced and important recommendation. We agree that the manuscript's positioning relative to existing SHAP-in-oncology literature was insufficiently specific, and that certain phrasings (e.g., 'conspicuously underexplored') could be read as overclaiming novelty. We have revised both the Introduction and Discussion to: (1) explicitly identify the specific gap this study addresses relative to prior SHAP-in-breast-cancer work, noting that multiple prior studies have applied SHAP to breast cancer classification but have not (a) systematically compared SHAP-derived feature rankings across multiple model architectures to identify algorithmically invariant versus model-dependent features; (b) integrated probability calibration analysis as a deployment-critical metric; or (c) contextualized findings within the operational constraints of LMIC diagnostic settings; (2) replaced broad novelty claims with precise, substantiated claims of contribution; and (3) softened language such as 'conspicuously underexplored' to 'insufficiently addressed in the context of [specific gap].' Two additional relevant references from the SHAP-in-clinical-ML literature have been added to contextualise our contributions more precisely.

Please see our revised Introduction, paragraph 4:

The phrase 'remains conspicuously underexplored' has been revised to: 'have received limited attention in the specific context of resource-limited settings, where computational parsimony and algorithmic transparency carry distinct operational weight. Prior SHAP-based analyses of breast cancer ML models have predominantly focused on single-model interpretability without systematic cross-model consensus analysis, probability calibration evaluation, or explicit contextualization for LMIC clinical deployment, dimensions that are central to the present study [21,22].’

Also in our revised Discussion 4.1, the contribution framing has been made more specific:

'The principal methodological contribution of this study, relative to prior SHAP-based analyses of breast cancer classifiers, are fourfold: (i) the introduction of cross-model SHAP consensus analysis, using arithmetic mean, geometric mean, and median rank alongside pairwise Spearman correlations, as a method for distinguishing algorithmically invariant biological signals from model-specific artifacts; (ii) integration of probability calibration analysis as a deployment-critical evaluation dimension alongside discriminative metrics; (iii) systematic mapping of SHAP-derived feature importance to established cytopathological grading criteria (Masood Classification); and (iv) contextualization of all findings within the specific operational constraints and clinical priorities of LMIC diagnostic settings, a combination not previously addressed in the breast cancer SHAP literature [33,34].

**Comment 2:** The paper proposes the concept of Translational Equity, but the discussion of its connotation is relatively abstract. Suggest clearly defining this concept in the discussion section.

**Response 2:** We thank Reviewer 2 for this conceptual observation. 'Translational Equity' is a framing concept introduced in this paper to capture the specific challenge that technically accurate AI systems may fail to achieve equitable impact if they are not designed with the interpretability, calibration, and infrastructural requirements of under-resourced settings. We have added a formal, definitional paragraph in Discussion section 4.3 that explicitly articulates the three-part construct of Translational Equity. We have also clarified that while the term is novel in our framing, it builds on established constructs in implementation science, responsible AI, and global health equity, which are cited accordingly.

Please see our revised Discussion 4.3 with added definitional paragraph:

**4.3. Implications for Resource-Limited Settings and Translational Equity**

We operationalize 'Translational Equity' as a three-component construct for evaluating AI-assisted diagnostics in resource-limited settings. The first component is technical accessibility: the AI system must be deployable within the computational and infrastructural constraints of the target setting, including standard hardware, offline functionality, and low inference latency. The second component is interpretive accessibility: the system must provide outputs that clinicians without specialist AI training can critically evaluate and meaningfully integrate,a requirement that calibrated probability outputs and SHAP-based explanations directly address. The third component is contextual alignment; the features identified as predictive must correspond to biological phenomena clinically recognizable and measurable within the diagnostic workflows of the target setting. A system that achieves high discriminative accuracy but fails on any one of these three components does not achieve Translational Equity, even on benchmark datasets. This distinction is critical because the primary bottleneck to equitable AI in global oncology is not the availability of algorithms but the absence of frameworks that make those algorithms trustworthy, usable, and contextually appropriate for the clinicians who most need them.

**Comment 3:** The results section extensively restates the data in the table, which is quite lengthy. Suggest simplifying to highlight only the most clinically and statistically significant findings, with specific values for readers to read from the table.

**Response 3:** We thank Reviewer 2 for this important presentation observation. We agree that the Results section contained excessive restatement of tabular data. We have substantially revised all Results subsections (3.1-3.6) to: (1) eliminate verbatim repetition of values available in Tables 1-3; (2) focus narrative text on the most clinically meaningful and statistically significant findings, directing readers to tables for specific values; and (3) integrate interpretive commentary where it adds analytic value beyond data recitation. The revised Results section is approximately 20% shorter than the original while retaining all substantive findings.

**Comment 4:** You set fixed hyperparameters for each model, but did not mention the tuning process. This may result in some models, especially XGBoost and SVM that are sensitive to hyperparameters, not performing at their best, affecting the fairness of the comparison. Clearly acknowledge this in the limitations section, or provide additional clarification on the principles on which you selected the current hyperparameters.

**Response 4:** We thank Reviewer 2 for this observation. Our comprehensive revision to Methods 2.4 and the Limitations section (4.6) fully addresses this concern. The hyperparameter selection procedure (two-stage approach: literature-informed initialization followed by 5-fold cross-validated grid search on the training partition) has been described in detail. The specific concern about XGBoost and SVM sensitivity has been explicitly acknowledged in the Limitations section. We note that the near-ceiling performance observed for XGBoost (AUC = 0.996) and SVM (AUC = 0.996) suggests that the selected hyperparameters are at least near-optimal for this dataset, though we acknowledge this cannot be guaranteed without exhaustive search.

This is a valid and consequential critique. We acknowledge that the absence of a clearly described hyperparameter optimization rationale represents a significant reporting gap. The hyperparameters reported in Supplementary Table S1 were not arbitrarily assigned; they were derived through a structured two-stage approach: (1) initial parameter ranges were informed by published algorithm-specific benchmarks and developer recommendations for structured tabular data; and (2) these ranges were refined through systematic grid search on the training partition using 5-fold cross-validation with AUC-ROC as the optimization criterion. For ensemble methods, the grid search encompassed n_estimators (100-500), max_depth (3-10), and learning_rate (0.01-0.20) for boosting algorithms. We have now fully described this hyperparameter optimization procedure in Section 2.4 of the revised manuscript. We have also explicitly acknowledged in Section 4.6 (Limitations) that the search space was bounded and that more exhaustive strategies (e.g., Bayesian optimization) may yield marginal additional gains, though the near-ceiling performance observed across models suggests these would be minimal for this dataset.

Please see our revised Methods 2.4, last paragraph:

Hyperparameter Configuration and Optimization: The hyperparameter configurations were determined through a two-stage approach. In stage one, initial parameter ranges were informed by published algorithm-specific benchmarks for structured tabular data and developer recommendations [28,29]. In stage two, these ranges were refined through systematic grid search conducted exclusively on the training partition (n = 455) using 5-fold stratified cross-validation with AUC-ROC as the primary optimization criterion. For boosting algorithms, the search space encompassed: n_estimators ∈ {100, 200, 300, 500}, max_depth ∈ {3, 5, 7, 10}, and learning_rate ∈ {0.01, 0.05, 0.10, 0.20}. The hold-out test set was strictly withheld from all hyperparameter selection procedures to prevent information leakage. Final configurations represent those maximizing cross-validated AUC while maintaining training-to-validation performance stability, as evidenced by the learning curve analysis (Supplementary Figure S1).

Also kindly see our revised Limitations 4.6:

Fourth, a key limitation of this study is the absence of true external validation. The hold-out test set utilized for performance evaluation was derived from the same WBCD dataset as the training partition and therefore does not fulfill TRIPOD-ML criteria for external validation, which requires evaluation on an independent dataset from a separate cohort, institution, or geographic context. The performance estimates reflect internal generalizability to this dataset's distributional properties rather than generalizability to cytological specimens from LMIC laboratories. External validation on independent FNAC datasets from LMIC facilities represents the single most critical methodological priority before any clinical deployment can be responsibly considered. We are currently pursuing collaborative partnerships with cytopathology laboratories in Sub-Saharan Africa and South Asia for prospective collection and analysis.

Fifth, while hyperparameter optimization was conducted through cross-validated grid search on the training partition, the bounded search space and single optimization criterion (AUC-ROC) may not have identified globally optimal configurations for all performance dimensions. For hyperparameter-sensitive algorithms such as XGBoost and SVM, more exhaustive strategies (e.g., Bayesian optimization) may yield marginal gains, though the near-ceiling performance observed suggests these would be clinically inconsequential for this dataset. Sixth, sensitivity analyses across alternative split ratios (e.g., 70:30, 75:25) were not formally conducted; however, the primary 10-fold cross-validation substantially mitigates split-ratio dependency concerns.

**Comment 5:** The arithmetic mean of Consensus Rank is very intuitive, but it is affected by extreme values. Consider supplementing or using geometric means or medians to enhance robustness against model-specific biases. Meanwhile, Spearman correlation coefficients between rankings should be reported.

**Response 5:** Reviewer 2 raises an excellent statistical point. The arithmetic mean of cross-model feature ranks is indeed susceptible to distortion by extreme values when rank scales differ substantially across models (e.g., when one model assigns a feature a rank of 28 while others assign it ranks of 13 and 15, the arithmetic mean is pulled upward disproportionately). We have made the following revisions to Table 3: (1) Added a Geometric Mean Consensus Rank column, which provides a more robust central tendency estimate by dampening the influence of extreme ranks while retaining sensitivity to consistent top-ranked features; (2) Added a Median Consensus Rank column as a non-parametric, outlier-robust alternative; and (3) Added a separate panel to Table 3 reporting pairwise Spearman rank correlation coefficients between the SHAP feature ranking vectors for XGBoost, Random Forest, and LightGBM. The computed Spearman correlations are: XGBoost vs. RF: ρ = 0.82, XGBoost vs. LightGBM: ρ = 0.86, and RF vs. LightGBM: ρ = 0.67, indicating that XGBoost and LightGBM (both gradient-boosted architectures) show higher mutual concordance than either does with Random Forest (a bagging-based architecture). These findings reinforce our cross-model consensus interpretation and have been discussed in Section 4.4.

Please see the added paragraph in our revised Discussion 4.4:

Pairwise Spearman correlation analysis revealed that XGBoost and LightGBM (both gradient-boosted architectures) demonstrated higher mutual concordance (ρ = 0.86) than either shared with Random Forest (XGBoost vs. RF: ρ = 0.82; RF vs. LightGBM: ρ = 0.67), suggesting that algorithmic architecture, specifically the boosting versus bagging paradigm, partially determines feature ranking concordance for this dataset. The geometric mean and median consensus ranks are fully consistent with arithmetic mean rankings for the top 5 features, while the divergence observed for lower-ranked features (e.g., compactness error: arithmetic rank 18 vs. geometric rank 17.6) reflects the sensitivity of the arithmetic mean to extreme RF rank values (rank 28), confirming the value of the robust consensus metrics.

**Comment 6:** The indicators such as AUC and F1 score in the table are given to four decimal places, and for medical applications, the difference in the first three decimal places is extremely small. It is recommended to keep the table to three significant figures to make it cleaner.

**Response 6:** We agree with Reviewer 2 that four decimal places convey spurious precision, particularly given the test set size (n = 114) where the confidence intervals around point estimates span several percentage points. All performance metrics in Table 2 and Table 3 have been rounded to three decimal places (three significant figures) in the revised manuscript. This also applies to CV accuracy and AUC standard deviations. The abstract and results text have been updated accordingly.

**Comment 7:** Some words have absolutization in their accuracy. More rigorous wording can be considered.

**Response 7:** We thank Reviewer 2 for this stylistic and epistemic observation. We have conducted a systematic review of the manuscript for absolutist or overconfident language and made the following representative revisions. The principle applied throughout is to replace declarative absolutism with appropriately hedged claims that acknowledge the scope limitations inherent in a single-dataset benchmark study.

Examplary representative language changes include:

(1) 'the three most influential predictive features' → 'the three most influential predictive features within this model on the WBCD'; (2) 'exceptional predictive performance' → 'strong predictive performance'; (3) 'definitively demonstrates' → 'provides evidence that'; (4) 'challenges the prevailing assumption' → 'calls into question the common assumption'; (5) 'near-ceiling discriminative performance' → 'near-ceiling discriminative performance for this dataset and evaluation conditions'; (6) 'these findings align precisely with' → 'these findings are consistent with'; (7) 'confirming' → 'supporting'; (8) 'ensure full computational reproducibility' → 'support full computational reproducibility'. All instances of absolutist language identified during review have been addressed in the tracked-changes version.

**Comment 8:** The article emphasizes that KNN achieves 100% recall, but also reasonably points out the comprehensive advantages of LR in the evaluation. The article mentions the three-stage implementation pathway, which is great. But it could be more realistic to add one or two sentences discussing the specific challenges that may be encountered in situations where resources are truly limited.

**Response 8:** We thank Reviewer 2 for this important recommendation to ground our implementation pathway in operational realism. The reviewer is correct that the three-stage pathway description was technically coherent but insufficiently candid about the structural barriers that resource-limited settings impose. We have added a dedicated paragraph in Section 4.3 explicitly addressing these challenges. Regarding KNN's perfect recall; we have also added a sentence noting that while KNN achieves 1.000 recall (zero missed malignancies), this comes at the cost of three false positives in the test set, a trade-off that may be acceptable in high-stakes screening contexts but warrants careful consideration in settings where confirmatory investigations (e.g., core biopsy, histopathology) require costly referral.

Please see added paragraph in our revised Discussion 4.3:

Honest acknowledgment of implementation barriers is essential to avoid creating unrealistic expectations. In truly resource-limited settings, the proposed three-stage pathway faces significant structural challenges beyond technical feasibility. First, retrospective validation (Stage i) requires access to locally annotated FNAC datasets, a resource absent in most LMIC cytopathology laboratories where digital archiving is rarely standardized. Second, integration into laboratory information management systems (Stage ii) presupposes electronic health record infrastructure that many peripheral facilities lack. Third, digitization of FNAC specimens requires calibrated digital microscopes or flatbed scanners, equipment often inaccessible at sub-district facilities. Fourth, regulatory pathways for AI-assisted diagnostics in most LMICs remain undefined, creating approval uncertainty. These challenges reinforce rather than diminish the importance of the Translational Equity framework: future development must engage implementation scientists, health systems specialists, and LMIC clinician communities from the earliest stages.

We trust that the revisions described above, implemented comprehensively in the revised manuscript, fully address the concerns of both reviewers and the Managing Editor. We remain committed to the highest standards of methodological rigor and transparent reporting and believe the revised manuscript is substantially strengthened. We look forward to the editorial team's further consideration.

Respectfully,

Oluwaseun Adebayo Bamodu, MD, MS, PhD

Corresponding Author | dr_bamodu@yahoo.com | ORCID: 0000-0001-8229-0408

On behalf of all co-authors
