# Supplementary material for: Explainable machine learning for breast cancer prediction in resource-constrained settings: A multi-algorithmic framework integrating shap-based transparency with clinical decision support
Source: PLOS Digit Health. 2026 Sep 11;5(9):e0001706. doi: 10.1371/journal.pdig.0001706 (PMC13568489; doi:10.1371/journal.pdig.0001706)
Supplement: Peer review history

# Peer review: Decision Letter 0

**DOI:** 10.1371/journal.pdig.0001706.r001

**Authors:** Amara Tariq, Jia-Lang Xu

22 May 2026

Response to Reviewers Revised Manuscript with Track Changes Manuscript **Journal Requirements:**

1. We ask that a manuscript source file is provided at Revision. Please upload your manuscript file as a .doc, .docx, .rtf or .tex.

2. Please upload separate figure files in .tif or .eps format. Also, remove the figures from your manuscript file but keep the legends.

For more information about figure files please see our guidelines:

https://journals.plos.org/digitalhealth/s/figures

https://journals.plos.org/digitalhealth/s/figures#loc-file-requirements

3. Please provide an Author Summary. This should appear in your manuscript between the Abstract (if applicable) and the Introduction, and should be 150–200 words long. The aim should be to make your findings accessible to a wide audience that includes both scientists and non-scientists. Sample summaries can be found on our website under Submission Guidelines:

https://journals.plos.org/digitalhealth/s/submission-guidelines#loc-parts-of-a-submission

4. We notice that your supplementary tables are included in the manuscript file. Please remove them and upload them with the file type 'Supporting Information'. Please ensure that each Supporting Information file has a legend listed in the manuscript after the references list.

**Additional Editor Comments (if provided):** **Reviewers' Comments:**

**Comments to the Author**

1. Does this manuscript meet PLOS Digital Health’s publication criteria?

Reviewer #1: Yes

Reviewer #2: Partly

**********

2. Has the statistical analysis been performed appropriately and rigorously?

Reviewer #1: Yes

Reviewer #2: No

**********

3. Have the authors made all data underlying the findings in their manuscript fully available (please refer to the Data Availability Statement at the start of the manuscript PDF file)?

The PLOS Data policy

Reviewer #1: Yes

Reviewer #2: Yes

**********

4. Is the manuscript presented in an intelligible fashion and written in standard English?

Reviewer #1: Yes

Reviewer #2: Yes

**********

**Reviewer #1:**  Thank you for inviting me to review the manuscript titled "Explainable Machine Learning for Breast Cancer Prediction in Resource-Constrained Settings:A Multi-Algorithmic Framework Intergrating SHAP-Based Transparency with Clinical Decision Support." The study addresses an important topic and demonstrates innovative use of explainable machine learning techniques. However, several methodological and reporting limitations should be addressed before the manuscript can be considered for acceptance.

A. The authors present fixed hyperparameters for all eight classifiers (Supplementary Table S1) without describing any systematic optimization procedure. It is unclear whether these configurations were obtained through grid search, Bayesian optimization, randomized search, or manual tuning. This omission is consequential because improper hyperparameter selection can result in optimistically biased performance estimates.

The authors should describe the hyperparameter selection strategy, including the validation method used.

B. The authors used an 80:20 train test split, which is common; however, the manuscript does not justify its selection given the size of the dataset.

The authors should briefly justify the choice of this split ratio and indicate whether alternative partitioning strategies were considered to conduct sensitivity analyses across different split ratios.

C. The manuscript refers to the 20% hold-out split dataset as "external validation." However, if both the training and test sets were derived from the same original dataset, this does not constitute true external validation. External validation requires evaluation on an independent dataset obtained from a different cohort, institution, or source. The absence of genuine external validation limits confidence in the model’s generalizability and real-world applicability. The authors should include an evaluation on an independent external dataset.

D. Can the authors confirm how they checked for the similarity between the training set and the hold-out dataset before the development of the classifier?

**Reviewer #2** : 1、It is recommended to clearly state the specific differences from existing SHAP research in the introduction and discussion sections, avoiding excessive claims of being first-time or blank.

2、The paper proposes the concept of Translational Equity, but the discussion of its connotation is relatively abstract. Suggest clearly defining this concept in the discussion section.

3、The results section extensively restates the data in the table, which is quite lengthy. Suggest simplifying to highlight only the most clinically and statistically significant findings, with specific values for readers to read from the table.

4、You set fixed hyperparameters for each model, but did not mention the tuning process. This may result in some models, especially XGBoost and SVM that are sensitive to hyperparameters, not performing at their best, affecting the fairness of the comparison. Clearly acknowledge this in the limitations section, or provide additional clarification on the principles on which you selected the current hyperparameters.

5、The arithmetic mean of Consensus Rank is very intuitive, but it is affected by extreme values. Consider supplementing or using geometric means or medians to enhance robustness against model specific biases. Meanwhile, Spearman correlation coefficients between rankings should be reported.

6、The indicators such as AUC and F1 score in the table are given to four decimal places, and for medical applications, the difference in the first three decimal places is extremely small. It is recommended to keep the table to three significant figures to make it cleaner.

7、Some words have absolutization in their accuracy. More rigorous wording can be considered。

8、The article emphasizes that KNN achieves 100% recall, but also reasonably points out the comprehensive advantages of LR in the evaluation. The article mentions the three-stage implementation pathway, which is great. But it could be more realistic to add one or two sentences discussing the specific challenges that may be encountered in situations where resources are truly limited.

**********

what does this mean?). If published, this will include your full peer review and any attached files.

**Do you want your identity to be public for this peer review?**  If you choose “no”, your identity will remain anonymous but your review may still be made public.

For information about this choice, including consent withdrawal, please see our Privacy Policy

Reviewer #1: No

Reviewer #2: No

**********

**Figure resubmission:**

**Reproducibility:**

To enhance the reproducibility of your results, we recommend that authors of applicable studies deposit laboratory protocols in protocols.io, where a protocol can be assigned its own identifier (DOI) such that it can be cited independently in the future. Additionally, PLOS ONE offers an option to publish peer-reviewed clinical study protocols. Read more information on sharing protocols at https://plos.org/protocols?utm_medium=editorial-email&utm_source=authorletters&utm_campaign=protocols

# Author comment: Author response to Decision Letter 1

**DOI:** 10.1371/journal.pdig.0001706.r002

1 Jun 2026

Attachment Submitted filename: Point-by-Point Response to Reviewers.docx

# Peer review: Decision Letter 1

**DOI:** 10.1371/journal.pdig.0001706.r003

**Authors:** Amara Tariq, Jia-Lang Xu

16 Aug 2026

Explainable Machine Learning for Breast Cancer Prediction in Resource-Constrained Settings: A Multi-Algorithmic Framework Integrating SHAP-Based Transparency with Clinical Decision Support

PDIG-D-26-00399R1

Dear Dr Bamodu,

We are pleased to inform you that your manuscript 'Explainable Machine Learning for Breast Cancer Prediction in Resource-Constrained Settings: A Multi-Algorithmic Framework Integrating SHAP-Based Transparency with Clinical Decision Support' has been provisionally accepted for publication in PLOS Digital Health.

Before your manuscript can be formally accepted you will need to complete some formatting changes, which you will receive in a follow-up email from a member of our team.

Please note that your manuscript will not be scheduled for publication until you have made the required changes, so a swift response is appreciated.

IMPORTANT: The editorial review process is now complete. PLOS will only permit corrections to spelling, formatting or significant scientific errors from this point onwards. Requests for major changes, or any which affect the scientific understanding of your work, will cause delays to the publication date of your manuscript.

If your institution or institutions have a press office, please notify them about your upcoming paper to help maximize its impact. If they'll be preparing press materials, please inform our press team as soon as possible -- no later than 48 hours after receiving the formal acceptance. Your manuscript will remain under strict press embargo until 2 pm Eastern Time on the date of publication. For more information, please contact digitalhealth@plos.org.

Thank you again for supporting Open Access publishing; we are looking forward to publishing your work in PLOS Digital Health.

Best regards,

Jia-Lang Xu

Academic Editor

PLOS Digital Health

***********************************************************

**Additional Editor Comments (if provided):**

During the review process, two reviewers provided recommendations for acceptance. Based on their positive evaluations, the manuscript is recommended for acceptance.

**Reviewer Comments (if any, and for reference):**

Reviewer's Responses to Questions

**Comments to the Author**

Reviewer #1: All comments have been addressed

Reviewer #3: All comments have been addressed

**********

publication criteria?

Reviewer #1: Yes

Reviewer #3: (No Response)

**********

3. Has the statistical analysis been performed appropriately and rigorously?

Reviewer #1: Yes

Reviewer #3: (No Response)

**********

4. Have the authors made all data underlying the findings in their manuscript fully available (please refer to the Data Availability Statement at the start of the manuscript PDF file)?

The PLOS Data policy

Reviewer #1: Yes

Reviewer #3: (No Response)

**********

5. Is the manuscript presented in an intelligible fashion and written in standard English?

Reviewer #1: Yes

Reviewer #3: (No Response)

**********

Reviewer #1: The authors have thoroughly addressed all of my comments on the hyperparameter optimization, dataset splitting, and similarity checks and have made substantial improvements to the manuscript. I have no further concerns. Thank you.

Reviewer #3: The authors have adequately addressed the concerns raised during the review process and provided satisfactory responses to the reviewers' comments. In my opinion, the manuscript has been substantially improved and is now suitable for publication. Therefore, I recommend its acceptance.

**********

what does this mean?). If published, this will include your full peer review and any attached files.

**Do you want your identity to be public for this peer review?**  If you choose “no”, your identity will remain anonymous but your review may still be made public.

For information about this choice, including consent withdrawal, please see our Privacy Policy

Reviewer #1: No

Reviewer #3: **Yes:** Pei-Chun Lin

**********
